# Primary sclerosing cholangitis and the risk of cancer, cardiovascular disease, and all-cause mortality: a systematic review and meta-analysis of cohort studies

**DOI:** 10.1038/s41598-021-90175-w

**Published:** 2021-05-20

**Authors:** Dagfinn Aune, Abhijit Sen, Teresa Norat, Elio Riboli, Trine Folseraas

**Affiliations:** 1 Department of Epidemiology and Biostatistics, School of Public Health, Imperial College London, St. Mary’s Campus, Norfolk Place, Paddington, London, W2 1PG UK; 2 Department of Nutrition, Bjørknes University College, Oslo, Norway; 3 Department of Endocrinology, Morbid Obesity and Preventive Medicine, Oslo University Hospital Ullevål, Oslo, Norway; 4 Unit of Cardiovascular and Nutritional Epidemiology, Institute of Environmental Medicine, Karolinska Institute, Stockholm, Sweden; 5 Department of Public Health and Nursing, Faculty of Medicine, Norwegian University of Science and Technology, Trondheim, Norway; 6 Center for Oral Health Services and Research (TkMidt), Trondheim, Norway; 7 Division of Surgery, Inflammatory Medicine and Transplantation, Department of Transplantation Medicine, Norwegian PSC Research Center, Oslo University Hospital Rikshospitalet, Oslo, Norway; 8 Institute of Clinical Medicine, Faculty of Medicine, University of Oslo, Oslo, Norway; 9 Division of Surgery, Inflammatory Medicine and Transplantation, Section for Gastroenterology, Department of Transplantation Medicine, Oslo University Hospital Rikshospitalet, Oslo, Norway

**Keywords:** Cancer, Cancer epidemiology, Cancer prevention, Gastrointestinal cancer

## Abstract

A diagnosis of primary sclerosing cholangitis (PSC) has been associated with increased risk of hepatobiliary cancers, colorectal cancer and all-cause mortality in several studies, while associations with cardiovascular disease have been inconsistent. We conducted a systematic review and meta-analysis of published cohort studies on the topic to summarize these associations. PubMed and Embase databases were searched up to January 13th, 2020. Cohort studies on PSC and risk of cancer, cardiovascular disease, or mortality were included. Summary relative risks (RRs) and 95% confidence intervals (95% CIs) were estimated using random effects models. The summary RR (95% CI) comparing persons with PSC to persons without PSC was 584.37 (269.42–1267.51, I^2^ = 89%, n = 4) for cholangiocarcinoma (CCA), 155.54 (125.34–193.02, I^2^ = 0%, n = 3) for hepatobiliary cancer, 30.22 (11.99–76.17, I^2^ = 0%, n = 2) for liver cancer, 16.92 (8.73–32.78, I^2^ = 88%, n = 4) for gastrointestinal cancer, 7.56 (2.42–23.62, I^2^ = 0%, n = 3) for pancreatic cancer, 6.10 (4.19–8.87, I^2^ = 14%, n = 7) for colorectal cancer (CRC), 4.13 (2.99–5.71, I^2^ = 80%, n = 5) for total cancer, 3.55 (2.94–4.28, I^2^ = 46%, n = 5) for all-cause mortality, and 1.57 (0.25–9.69, I^2^ = 79%, n = 2) for cardiovascular disease. Strong positive associations were observed between PSC and risk of CCA, hepatobiliary cancer, liver cancer, gastrointestinal cancer, pancreatic cancer, CRC, total cancer, and all-cause mortality, but not for cardiovascular disease.

## Introduction

Primary sclerosing cholangitis (PSC) is a chronic disease of the biliary tree characterized by progressive inflammation, and fibrotic stricturing of the bile ducts^1^. In absence of effective medical treatment, the majority of patients will gradually develop liver cirrhosis and end-stage liver disease, where liver transplantation represents the only curative option for patients with end-stage disease. PSC is a rare disease with an incidence of 0.4–0.7 cases per 100,000 in the UK^2^ and a prevalence of 3.9–16.2 per 100,000^1^. Little is known about the causes of the disease, but genetic risk factors suggest a role of immune-mediated mechanisms in the disease development as there is genetic overlap with autoimmune diseases, such as type 1 diabetes, coeliac disease, rheumatoid arthritis, sarcoidosis and psoriasis^3^. Inflammation in the colon is a predominant risk factor for PSC with concomitant inflammatory bowel disease being reported in up to 60–80% of PSC patients^1^. Higher intake of coffee has been found to be inversely associated with PSC^4,5^, which is partly consistent with the protective effect of coffee on liver cirrhosis and liver cancer^6^. Smoking has shown a paradoxical inverse association with risk of PSC^4,7–9^, however, it is unclear if this finding is causal as most of the available studies are of retrospective case–control study design, which may be prone to recall and selection bias.

Prospective cohort studies have suggested strong positive associations between PSC and increased risk of several gastrointestinal cancers with extreme relative risks (RRs) in the range of 200–1560 for cholangiocarcinoma^10–13^, 117–177 for hepatobiliary cancer^11,14,15^, 78 for gallbladder cancer^13^, and 22–42 for liver cancer^2,13^. In addition, several studies have suggested increased risk of gastrointestinal cancer overall^11,14,16^, pancreatic cancer^13,14,17^, colorectal cancer^11–14,17,18^, total cancer^2,11,13,14^ as well as all-cause mortality^2,11–13,15^. A potential association between PSC and cardiovascular disease (CVD) has been more unclear with only a limited number of studies published on the subject^15,19^. However, the available data on risk of cancer, cardiovascular disease and mortality in PSC have to our knowledge not yet been summarized. Therefore, to provide more stable estimates of risk of cancer, cardiovascular disease and mortality in patients with PSC we conducted a systematic review and meta-analysis of the published cohort studies on the topic.

## Material and methods

### Search strategy

We searched the PubMed and Embase databases up to January 13th, 2020 for eligible studies. The search terms used are found in the Supplementary Text. In addition, we searched the reference lists of the identified publications for further studies. We followed standard PRISMA criteria for reporting meta-analyses^20^.

### Study selection

We included published retrospective and prospective cohort studies that investigated the association between PSC and the risk of cancer, cardiovascular disease and all-cause mortality. Estimates of the relative risk (RR) had to be available with the 95% confidence intervals (CIs) in the publication. A list of the excluded studies and exclusion reasons is found in Supplementary Table 1.

### Data extraction

The following data were extracted from each study: The first author’s last name, publication year, country where the study was conducted, study period, sample size, number of cases, age, exposure (PSC) and subgroups (e.g., incidence vs. mortality, disease subtypes or excluding early follow-up), comparison, RRs and 95% CIs for patients with PSC compared to persons without the condition, and variables adjusted for in the analysis. DA extracted the data and AS double-checked the data for accuracy.

### Statistical methods

We calculated summary RRs (95% CIs) for the association between PSC and risk of cancer, cardiovascular disease, and mortality using the random-effects model by DerSimonian and Laird^21^ which takes into account both within and between study variation (heterogeneity). The average of the natural logarithm of the RRs was estimated and the RR from each study was weighted using random effects weights.

Heterogeneity between studies was evaluated using Q and I^2^ statistics^22^. Cochran’s Q is calculated as the weighted sum of squared differences between individual study effects and the pooled effects across studies, with weights being those in the pooling method. I^2^ is a measure of how much of the heterogeneity that is due to between study variation rather than chance. I^2^-values of 25%, 50% and 75% indicates low, moderate and high heterogeneity respectively. The Newcastle–Ottawa scale was used to assess the quality of the included studies^23^.

Publication bias was assessed using Egger’s test^24^ and Begg–Mazumdar’s test^25^. Egger’s test is a statistical test for asymmetry of the funnel plot and is a linear regression in which the standard normal deviate (estimate divided by its standard error) is regressed against its precision (inverse of standard deviate). Begg’s test is a test of the interdependence of variance and effect size using Kendall’s method. Sensitivity analyses were carried out excluding one study at a time from the analyses to assess whether the summary estimates were driven by a very large study or a study with an extreme results. The statistical analyses were conducted using the software package Stata, version 13.0 software (StataCorp, Texas, US).

## Results

**Figure 1:**
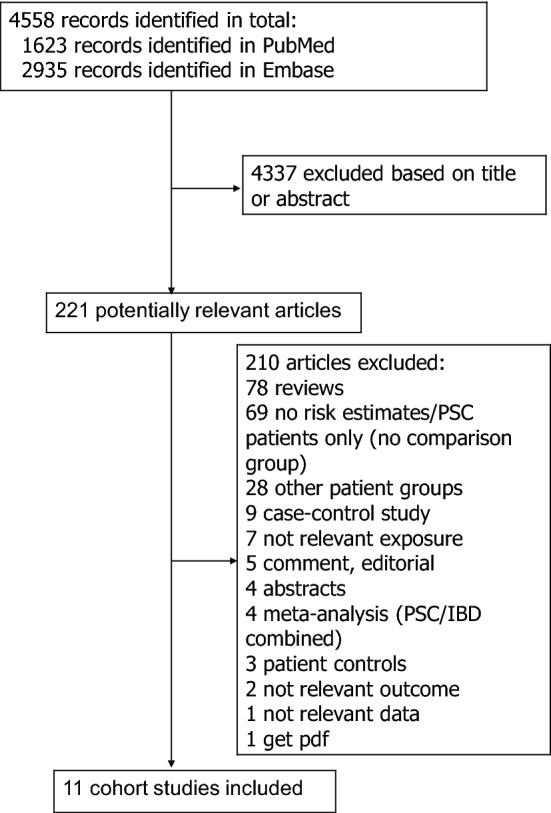
Flow-chart of study selection.

We identified eleven cohort studies that were included in the meta-analysis^2,10–19^ (Fig. 1, Supplementary Tables 2–10). Study characteristics are shown in Supplementary Tables 2–10. Eight studies were conducted in Europe, two in the USA and one in New Zealand. Results for outcomes with too few studies for meta-analyses are shown in Supplementary Table 11.

**Figure 2:**
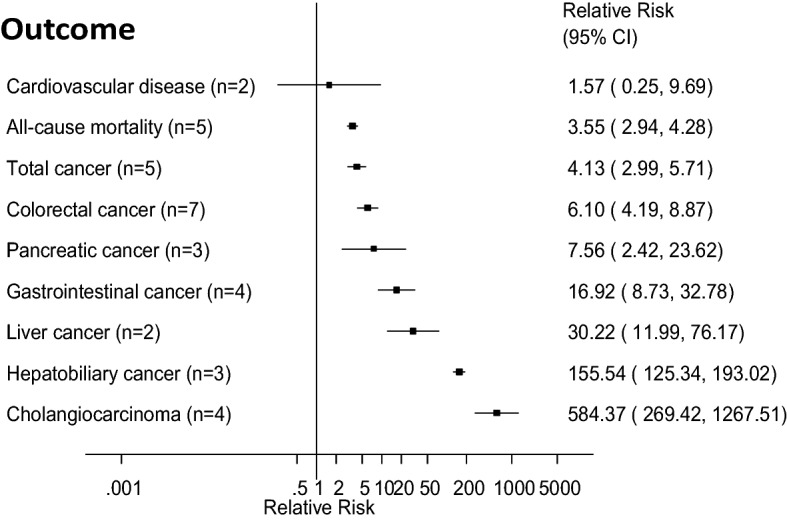
Primary sclerosing cholangitis and cancer, cardiovascular disease and all-cause mortality. Summary relative risks for each outcome are represented by the squares and the 95% confidence intervals are represented by the lines through the squares (n = number of studies).

**Figure 3:**
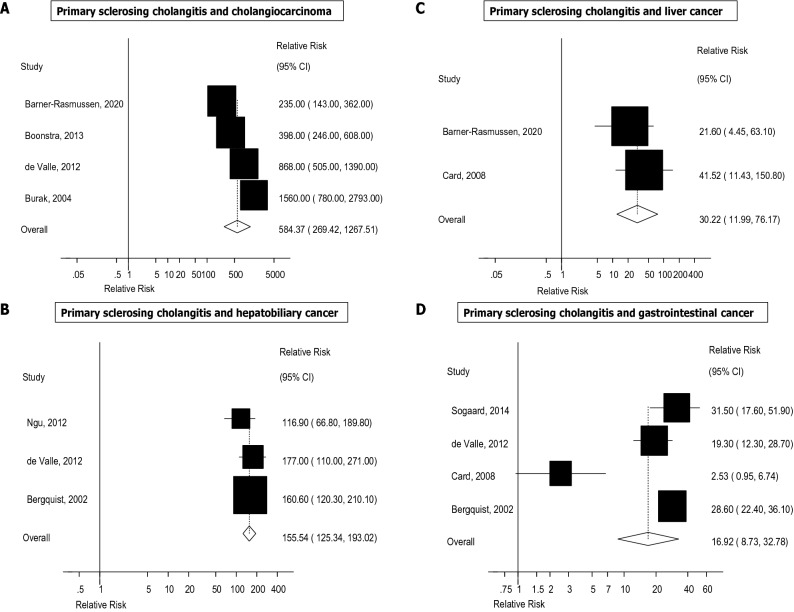
Primary sclerosing cholangitis and cholangiocarcinoma, hepatobiliary cancer, liver cancer and gastrointestinal cancer overall.

**Figure 4:**
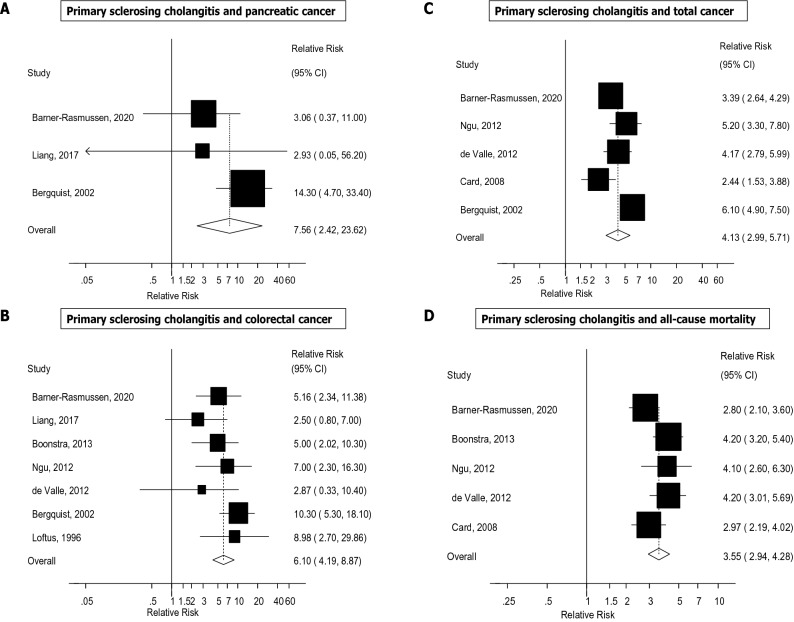
Primary sclerosing cholangitis and pancreatic cancer, colorectal cancer, total cancer and all-cause mortality.

**Figure 5:**
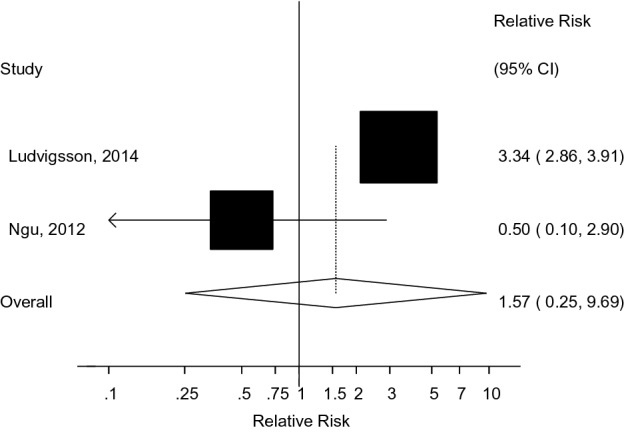
Primary sclerosing cholangitis and cardiovascular disease.

The summary RRs (95% CIs) across outcomes is provided in Fig. 2. The summary RR (95% CI) comparing persons with PSC with persons without PSC was 584.37 (95% CI 269.42–1267.51, I^2^ = 89%, n = 4) for cholangiocarcinoma^10–13^ (Fig. 3A), 155.54 (95% CI 125.34–193.02, I^2^ = 0%, n = 3) for hepatobiliary cancer (Fig. 3B)^2,11,13–15^, 30.22 (95% CI 11.99–76.17, I^2^ = 0%, n = 2) for liver cancer (Fig. 3C), 16.92 (95% CI 8.73–32.78, I^2^ = 88%, n = 4) for gastrointestinal cancer^2,11,14,16^ (Fig. 3D), 7.56 (95% CI 2.42–23.62, I^2^ = 0%, n = 3) for pancreatic cancer^13,14,17^ (Fig. 4A), 6.10 (95% CI 4.19–8.87, I^2^ = 14%, n = 7) for colorectal cancer^11–15,17,18^ (Fig. 4B), 4.13 (95% CI 2.99–5.71, I^2^ = 80%, n = 5) for total cancer^2,11,13,14,16^ (Fig. 4C), 3.55 (95% CI 2.94–4.28, I^2^ = 46%, n = 5) for all-cause mortality^2,11–13,15^ (Fig. 4D), and 1.57 (95% CI 0.25–9.69, I^2^ = 79%, n = 2) for CVD^15,19^ (Fig. 5). There was little indication of publication bias with Egger's test with the exception of the analysis of liver cancer (Table 1), however, the number of studies was small.

**Table 1:** Summary relative risks and 95% confidence intervals for the association between primary sclerosing cholangitis and cancer and all-cause mortality.

Outcome	N	Cases	Participants	RR (95% CI)	I^2^	P_heterogeneity_	Egger	References
Cholangiocarcinoma	4	92	1582	584.37 (269.42–1267.51)	89%	< 0.0001	0.15	^10–13^
Hepatobiliary cancer	3	46	2720	155.54 (125.34–193.02)	0%	0.47	0.67	^11,14,15^
Liver cancer	2	56	1236	30.22 (11.99–76.17)	0	0.49	–	^2,13^
Gastrointestinal cancer	4	115	3421	16.92 (8.73–32.78)	87.7	< 0.0001	0.22	^2,11,14,16^
Pancreatic cancer	3	15	2736	7.56 (2.42–23.62)	28.6%	0.25	0.42	^13,14,17^
Colorectal cancer	7	71	3774	6.10 (4.19–8.87)	14.0	0.32	0.17	^11–15,17,18^
Total cancer	5	336	3956	4.13 (2.99–5.71)	80.3	< 0.0001	0.44	^2,11,13–15^
All-cause mortality	5	477	3942	3.55 (2.94–4.28)	45.7	0.12	0.68	^2,11–13,15^
Cardiovascular disease	2	971	7106	1.57 (0.25–9.69)	79.4	0.03	–	^15,19^

### Other cancers

For several cancers there was only one study available and meta-analyses were therefore not possible. A study from Finland reported a RR of 78.3 (95% CI 21.3–200) for gallbladder cancer^13^. A study from New Zealand reported RRs of 117.6 (95% CI 24.2–343.6) for nonmelanoma skin cancer, 4.3 (95% CI 0.5–15.4) for lung cancer, 4.1 (95% CI 0.5–14.8) for breast cancer, 1.2 (95% CI < 0.1–6.7) for prostate cancer, and 0.8 (95% CI < 0.1–4.6) for renal cell carcinoma^15^. A Swedish study reported a RR of 2.2 (95% CI 0.1–12.5) for stomach cancer^14^.

### Sensitivity analyses and study quality

We conducted sensitivity analyses removing one study at a time from each analysis, however, the summary estimates were largely similar across these sensitivity analyses (Supplementary Figs. 1–6). The mean (median) study quality was 6.5 (6.0) (Supplementary Table 12).

## Discussion

To our knowledge this is the first meta-analysis of the relation between PSC and the risk of cancer, cardiovascular disease and all-cause mortality. Strong positive associations were observed between a diagnosis of PSC and the risk of several gastrointestinal cancers as well as all-cause mortality with RRs of 584 for cholangiocarcinoma, 156 for hepatobiliary cancer, 30 for liver cancer, 17 for gastrointestinal cancer, 7.6 for pancreatic cancer, 6.1 for colorectal cancer, 4.1 for total cancer and 3.6 for all-cause mortality. No association was found for cardiovascular disease, but the limited number of studies precludes any firm conclusions.

The meta-analysis has some limitations which need to be mentioned. The number of studies included in each analysis was low, and therefore we cannot exclude the possibility that some of these findings may slightly change when additional studies accrue. In most of the sensitivity analyses excluding one study at a time, no single study accounted for the observed associations, and strong positive associations remained across most analyses. For several cancers we only identified one study which met our inclusion criteria and we were therefore not able to conduct analyses for these cancers. This includes gallbladder cancer, which is known to be strongly related to PSC^13,26^. In addition, we were not able to conduct meaningful subgroup analyses and the tests for publication bias need to be interpreted with caution as they are less reliable when fewer studies are included in the analyses. Heterogeneity was observed in several analyses, however, all studies were consistent in finding increased risk and the heterogeneity appears to be driven by differences in the strength of the association rather than differences due to the presence or absence of an association between studies. Given the low incidence of PSC, little is known about the risk factors for the disease and there is potential for confounding of the association by potentially unknown risk factors. Most of the included studies reported results as standardized incidence ratios or standardized mortality ratios and adjusted only for age and sex, thus residual confounding is possible. The few available retrospective case–control studies indicate that coffee^4,5^ and smoking^4,7–9^ may be associated with lower risk of PSC. Although coffee intake has been associated with reduced risk of liver cancer (RR = 0.5)^6^ this association is not sufficiently strong to fully account for the RR of 30 for liver cancer among patients with PSC. On the other hand, if smoking is truly protective for PSC this would most likely lead to negative confounding for cancer and mortality as smoking is strongly positively associated with both these outcomes. One study which additionally adjusted for smoking status found associations which were slightly weaker than the remaining studies, however, within that study the smoking-adjusted hazard ratios were slightly stronger than the unadjusted hazard ratios^2^. This suggests that other differences perhaps due to study design and/or analyses could have contributed to the slight difference in results between this study and the remaining studies. Considering the very strong observed associations as well as the specificity of the associations (particularly between PSC and liver cancer and CCA), it seems perhaps less likely that confounding by lifestyle factors could fully account for these strong associations. However, we cannot exclude the possibility that other shared risk factors (e.g., genetic, infectious agents) could explain a proportion of the associations. The prevalence of inflammatory bowel disease (IBD) is high in PSC^27^ and it is possible that the association between PSC and CRC could be partly explained by IBD. However, previous studies suggested that patients with both PSC and IBD had a 3.4-fold increase in CRC risk compared to patients with only IBD, suggesting that PSC increases CRC risk beyond what is solely due to IBD^28^. One study excluded cases of small duct PSC from the analyses^10^, however, exclusion of that study (or any other study) did not materially alter the observed associations. Misdiagnosis of common bile duct cholangiocarcinoma as pancreatic head cancer could be a potential explanation for the association with pancreatic cancer.

Several biological mechanisms or pathways may explain why PSC is associated with increased risk of cancer and mortality. Pro-oncogenic processes resulting from chronic biliary inflammation and cholestasis with accumulation of bile acids is suspected to contribute to the development of CCA, possibly through induction of nitric oxide synthase and nitric oxide generation, which promotes oxidative DNA damage and inhibits DNA repair^29^ and could result in oncogenic mutations. Some reports have shown mutations in loci with tumor suppressor function, such as p16^INK4a^, both in PSC cases without cholangiocarcinoma and PSC cases with cholangiocarcinoma^30^. Mutations, homozygous deletions or inactivation by methylation in p16^INK4a^ have also been observed across several cancers, e.g., pancreatic, esophageal, bladder and liver cancer and familial melanoma^31,32^, homozygous deletions in bladder, kidney, pancreas and ovary tumors^33–35^, and inactivation through methylation in cancers of the breast, prostate, head and neck, and hepatocellular carcinomas^36–39^. The ErbB receptor kinase family are strong mediators of development of sporadic CCA, and interact with COX-2, interleukin-6, VEGF and Met activating not only their own pathway, but enhancing that of others^40^. It has been shown that ErbB2 is increased in patients with PSC as well as hepatolithiasis (which is also a risk factor for cholangiocarcinoma)^41^.

Progressive inflammation and scarring of the liver parenchyma in more advanced PSC may increase risk of liver cancer development through upregulation of proliferative signaling pathways^42^, and chronic intestinal inflammation may increase risk for CRC in the 60–80% of patients with PSC that have concomitant inflammatory bowel disease^27^. PSC is known to be related to pancreatitis^43^, which is an important risk factor for pancreatic cancer^44^.

The current findings are concerning given the strength of the observed associations and the rather poor survival rates for several of these cancers, particularly in CCA, liver cancer and pancreatic cancer. The results underscore the need for effective cancer surveillance in this patient group, as well as identification of markers for early diagnosis of cancer. In addition, implementation of primary prevention strategies by identification of potentially modifiable risk factors or pharmaceutical targets involved in cancer development are warranted.

In conclusion, this meta-analysis suggest that PSC is a very strong risk factor for CCA and liver cancer, but is also strongly associated with total gastrointestinal, pancreatic, colorectal, and total cancer risk as well as all-cause mortality. Although further studies are needed for a more comprehensive assessment of the association across a larger number of outcomes and with adjustments for additional confounding factors, the current findings clearly indicate that PSC patients need to be followed up closely with regard to cancer risk.

## Supplementary Information

Supplementary Information.
