# Supplementary material for: Primary sclerosing cholangitis and the risk of cancer, cardiovascular disease, and all-cause mortality: a systematic review and meta-analysis of cohort studies
Source: Sci Rep. 2021 May 20;11:10646. doi: 10.1038/s41598-021-90175-w (PMC8137938; doi:10.1038/s41598-021-90175-w)
Supplement: Supplementary file 1 — Supplementary Information. [file 41598_2021_90175_MOESM1_ESM.pdf]

Supplementary material for:

Dagfinn Aune, Abhijit Sen, Teresa Norat, Elio Riboli, Trine Folseraas. Primary sclerosing cholangitis and the risk of cancer, cardiovascular disease, and all-cause mortality: a systematic review and meta-analysis of cohort studies.

Scientific Reports 2021 DOI: 10.1038/s41598-021-90175-w

Supplementary text: PubMed search strategy

1) "primary sclerosing cholangitis" OR "sclerosing cholangitis"

2) Oral OR pharyngeal OR pharynx OR oropharyngeal OR oropharynx OR hypopharyngeal OR hypopharynx OR nasal OR paranasal sinus OR Nasopharyngeal OR nasopharynx OR Laryngeal OR larynx OR Esophageal OR esophagus OR oesophageal OR oesophagus OR “Upper aerodigestive tract” OR “head and neck” OR Lung OR respiratory OR Stomach OR gastric OR “Small intestinal” OR “small intestine” OR “small bowel” OR Pancreatic OR pancreas OR Liver OR hepatocellular OR Gallbladder OR “Bile duct” OR Colon OR rectal OR rectum OR colorectal OR colorectum OR “large bowel” OR Breast OR mammary OR Ovarian OR ovary OR Endometrial OR endometrium OR “corpus uteri” OR uterine OR Cervical OR cervix OR Prostate OR Testicular OR testes OR penis OR penile OR Kidney OR renal OR “renal cell” OR adrenal OR Bladder OR urothelial OR “urinary tract” OR Brain OR Thyroid OR anal

3) Cancer OR carcinoma OR neoplasm OR tumor OR tumour

4) Cholangiocarcinoma OR lymphoma OR non-Hodgkin’s lymphoma OR non-Hodgkin lymphoma OR Hodgkin’s lymphoma OR Hodgkin lymphoma OR Hodgkin disease OR leukemia OR myeloma OR melanoma OR glioma OR meningioma OR sarcoma

5) “coronary heart disease” OR “heart disease” OR “ischemic heart disease” OR “ischaemic heart disease” OR “coronary artery disease” OR “myocardial infarction” OR “angina pectoris” OR “heart failure” OR "atrial fibrillation" OR "sudden cardiac death" OR "aortic aneurysm" OR "venous thromboembolism" OR "pulmonary embolism" OR stroke OR “ischemic stroke” OR “haemorrhagic stroke” OR “cerebrovascular disease” OR “cardiovascular disease” OR cancer OR “total cancer” OR neoplasm OR mortality OR “all-cause mortality” OR “total mortality” OR survival OR death

6) "case-control" OR cohort OR prospective OR longitudinal OR retrospective OR "follow-up" OR "cross-sectional" OR "hazard ratio" OR "hazard ratios" OR "relative risk" OR "relative risks" OR "incidence rate ratio" OR "incidence rate ratios" OR "odds ratio" OR odds ratios OR incidence

7) 2 AND 3

8) 3 OR 4 OR 5 OR 7

9) 1 AND 8 AND 6

Embase search strategy

1) primary sclerosing cholangitis OR sclerosing cholangitis

2) primary sclerosing cholangitis/ OR sclerosing cholangitis/

3) Oral OR pharyngeal OR pharynx OR oropharyngeal OR oropharynx OR hypopharyngeal OR hypopharynx OR nasal OR paranasal sinus OR Nasopharyngeal OR nasopharynx OR Laryngeal OR larynx OR Esophageal OR esophagus OR oesophageal OR oesophagus OR Upper aerodigestive tract OR head and neck OR Lung OR respiratory OR Stomach OR gastric OR Small intestinal OR small intestine OR small bowel OR Pancreatic OR pancreas OR Liver OR hepatocellular OR Gallbladder OR Bile duct OR Colon OR rectal OR rectum OR colorectal OR colorectum OR large bowel OR Breast OR mammary OR Ovarian OR ovary OR Endometrial OR endometrium OR corpus uteri OR uterine OR Cervical OR cervix OR Prostate OR Testicular OR testes OR penis OR penile OR Kidney OR renal OR renal cell OR adrenal OR Bladder OR urothelial OR urinary tract OR Brain OR Thyroid OR anal

4) Oral/ OR pharyngeal/ OR pharynx/ OR oropharyngeal/ OR oropharynx/ OR hypopharyngeal/ OR hypopharynx/ OR nasal/ OR paranasal sinus/ OR Nasopharyngeal/ OR nasopharynx/ OR Laryngeal/ OR larynx/ OR Esophageal/ OR esophagus/ OR oesophageal/ OR oesophagus/ OR Upper aerodigestive tract/ OR Lung/ OR respiratory/ OR Stomach/ OR gastric/ OR Small intestinal/ OR small intestine/ OR small bowel/ OR Pancreatic/ OR pancreas/ OR Liver/ OR hepatocellular/ OR Gallbladder/ OR Bile duct/ OR Colon/ OR rectal/ OR rectum/ OR colorectal/ OR colorectum/ OR large bowel/ OR Breast/ OR mammary/ OR Ovarian/ OR ovary/ OR Endometrial/ OR endometrium/ OR corpus uteri/ OR uterine/ OR Cervical/ OR cervix/ OR Prostate/ OR Testicular/ OR testes/ OR penis/ OR penile/ OR Kidney/ OR renal/ OR renal cell/ OR adrenal/ OR Bladder/ OR urothelial/ OR urinary tract/ OR Brain/ OR Thyroid/ OR anal/

5) Cancer OR carcinoma OR neoplasm OR tumor OR tumour

6) Cancer/ OR carcinoma/ OR neoplasm/ OR tumor/ OR tumour/

7) Cholangiocarcinoma OR lymphoma OR non-Hodgkins lymphoma OR non-Hodgkin lymphoma OR Hodgkins lymphoma OR Hodgkin lymphoma OR Hodgkin disease OR leukemia OR myeloma OR melanoma OR glioma OR meningioma OR sarcoma

8) Cholangiocarcinoma/ OR lymphoma/ OR non-Hodgkins lymphoma/ OR non-Hodgkin lymphoma/ OR Hodgkins lymphoma/ OR Hodgkin lymphoma/ OR Hodgkin disease/ OR leukemia/ OR myeloma/ OR melanoma/ OR glioma/ OR meningioma/ OR sarcoma/

9) coronary heart disease OR heart disease OR ischemic heart disease OR ischaemic heart disease OR coronary artery disease OR myocardial infarction OR angina

pectoris OR heart failure OR atrial fibrillation OR sudden cardiac death OR aortic aneurysm OR venous thromboembolism OR pulmonary embolism OR stroke OR ischemic stroke OR haemorrhagic stroke OR cerebrovascular disease OR cardiovascular disease OR cancer OR total cancer OR neoplasm OR mortality OR all-cause mortality OR total mortality OR survival OR death

10) coronary heart disease/ OR heart disease/ OR ischemic heart disease/ ORischaemic heart disease/ OR coronary artery disease/ OR myocardial infarction/ OR angina pectoris/ OR heart failure/ OR atrial fibrillation/ OR sudden cardiac death/ OR aortic aneurysm/ OR venous thromboembolism/ OR pulmonary embolism/ OR stroke/ OR ischemic stroke/ ORhaemorrhagic stroke/ OR cerebrovascular disease/ OR cardiovascular disease/ OR cancer/ OR total cancer/ OR neoplasm/ OR mortality/ OR all-cause mortality/ OR total mortality/ OR survival/ OR death/

11) case-control OR cohort OR prospective OR longitudinal OR retrospective OR follow-up OR cross-sectional OR hazard ratio OR hazard ratios OR relative risk OR relative risks OR incidence rate ratio OR incidence rate ratios OR odds ratio OR odds ratios OR incidence

12) 1 OR 2

13) 3 OR 4

14) 5 OR 6

15) 13 AND 14

16) 7 OR 8 OR 9 OR 10 OR 14 OR 15

17) 12 AND 16 AND 11

Supplementary Table 1. List of excluded studies and exclusion reasons

**Exclusion reason**	**Reference number**
Abstract	(1-4)
Case-control study	(5-13)
Comment	(14-17)
Editorial	(18)
Meta-analysis (IBD and PSC combined)	(19-22)
No risk estimates	(23-71)
Not relevant data	(72)
Not relevant exposure	(73-79)
Not relevant outcome	(80;81)
Patient controls	(82-84)
Patient populations	(85-112)
PSC patients only (no control/comparison group)	(113-132)
Review	(133-210)

Reference List

1. Barner-Rasmussen N, Farkkila MA. Survival of primary sclerosing cholangitis: A population-based study in Finland. United European Gastroenterology Journal Conference: 26th United European Gastroenterology Week, UEG 2018;October.

2. Simionato F, Cazzagon N, Llovet LP et al. Hepatobiliary and non-hepatobiliary malignancies in psc patients from Southern Europe: A comparative study in two European centers. Hepatology Conference: 69th Annual Meeting of the American Association for the Study of Liver Diseases, AASLD 2018;October.

3. Carbone M, Kodra Y, Rocchetti A et al. Incidence, prevalence and mortality of primary sclerosing cholangitis in Italy: A population-based study. Journal of Hepatology Conference: The International Liver Congress 2019;April.

4. Wu K, Connell W, Bell S et al. Clinical outcomes and management of patients with concomitant primary sclerosing cholangitis and inflammatory bowel disease. Journal of Gastroenterology and Hepatology Conference: Gastroenterological Society of Australia,

GESA and Australian Gastroenterology Week, AGW "Gut Matters" Australia 33 (Supplement 2) (pp 114), 2018;September.

5. Bansal P, Sonnenberg A. Risk factors of colorectal cancer in inflammatory bowel disease. Am J Gastroenterol 1996;91:44-8.

6. Bergquist A, Glaumann H, Persson B, Broome U. Risk factors and clinical presentation of hepatobiliary carcinoma in patients with primary sclerosing cholangitis: a case-control study. Hepatology 1998;27:311-6.

7. Leidenius M, Hockersted K, Broome U et al. Hepatobiliary carcinoma in primary sclerosing cholangitis: a case control study. J Hepatol 2001;34:792-8.

8. Bjornsson E, Angulo P. Cholangiocarcinoma in young individuals with and without primary sclerosing cholangitis. Am J Gastroenterol 2007;102:1677-82.

9. Zhou Z, Nie S-D, Jiang B, Wang J, Lv P. Risk factors for extrahepatic cholangiocarcinoma: A case-control study in China. European Journal of Cancer Prevention 2019;28:254-7.

10. McGee EE, Castro FA, Engels EA et al. Associations between autoimmune conditions and hepatobiliary cancer risk among elderly US adults. International Journal of Cancer 2019;144:707-17.

11. Rutter M, Saunders B, Wilkinson K et al. Severity of inflammation is a risk factor for colorectal neoplasia in ulcerative colitis. Gastroenterology 2004;126:451-9.

12. Velayos FS, Loftus EV, Jr., Jess T et al. Predictive and protective factors associated with colorectal cancer in ulcerative colitis: A case-control study. Gastroenterology 2006;130:1941-9.

13. Biewenga M, Van Den BA, Van BH, van HB. Characteristics and outcome of autoimmune hepatitis-primary biliary cholangitis and autoimmune hepatitis-primary sclerosing cholangitis overlap patients compared to autoimmune hepatitis patients. Journal of Hepatology Conference: The International Liver Congress 2019;April.

14. Helper DJ. PSC in UC: is it or is it not a risk factor for the development of neoplasia? Inflamm Bowel Dis 2000;6:61-2.

15. Stiehl A. Primary sclerosing cholangitis: neoplastic potential in bile ducts, colon and the pancreas? J Hepatol 2002;36:433-4.

16. Kingham JG, Kochar N, Gravenor MB. Incidence, clinical patterns, and outcomes of primary sclerosing cholangitis in South Wales, United Kingdom. Gastroenterology 2004;126:1929-30.

17. Shariff MI, Maggs J, Heneghan MA. Mortality and the risk of malignancy in autoimmune liver diseases: a population-based study in Canterbury, New Zealand. Hepatology 2013;57:1677.

18. Sokol H, Chazouilleres O. Colorectal neoplasia in PSC-IBD patients: are times changing? Liver Int 2012;32:175-6.

19. Soetikno RM, Lin OS, Heidenreich PA, Young HS, Blackstone MO. Increased risk of colorectal neoplasia in patients with primary sclerosing cholangitis and ulcerative colitis: a meta-analysis. Gastrointest Endosc 2002;56:48-54.

20. Singh S, Edakkanambeth VJ, Loftus EV, Jr., Talwalkar JA. Incidence of colorectal cancer after liver transplantation for primary sclerosing cholangitis: a systematic review and meta-analysis. Liver Transpl 2013;19:1361-9.

21. Zheng HH, Jiang XL. Increased risk of colorectal neoplasia in patients with primary sclerosing cholangitis and inflammatory bowel disease: a meta-analysis of 16 observational studies. Eur J Gastroenterol Hepatol 2016;28:383-90.

22. Komaki Y, Komaki F, Micic D, Ido A, Sakuraba A. Risk of colorectal cancer in chronic liver diseases: a systematic review and meta-analysis. Gastrointest Endosc 2017;86:93-104.

23. Wiesner RH, Grambsch PM, Dickson ER et al. Primary sclerosing cholangitis: natural history, prognostic factors and survival analysis. Hepatology 1989;10:430-6.

24. Higashi H, Yanaga K, Marsh JW, Tzakis A, Kakizoe S, Starzl TE. Development of colon cancer after liver transplantation for primary sclerosing cholangitis associated with ulcerative colitis. Hepatology 1990;11:477-80.

25. Ismail T, Angrisani L, Powell JE et al. Primary sclerosing cholangitis: surgical options, prognostic variables and outcome. Br J Surg 1991;78:564-7.

26. Wiesner RH, Porayko MK, Dickson ER et al. Selection and timing of liver transplantation in primary biliary cirrhosis and primary sclerosing cholangitis. Hepatology 1992;16:1290-9.

27. Abu-Elmagd KM, Malinchoc M, Dickson ER et al. Efficacy of hepatic transplantation in patients with primary sclerosing cholangitis. Surg Gynecol Obstet 1993;177:335-44.

28. Abu-Elmagd KM, Selby R, Iwatsuki S et al. Cholangiocarcinoma and sclerosing cholangitis: clinical characteristics and effect on survival after liver transplantation. Transplant Proc 1993;25:1124-5.

29. Gurbuz AK, Giardiello FM, Bayless TM. Colorectal neoplasia in patients with ulcerative colitis and primary sclerosing cholangitis. Dis Colon Rectum 1995;38:37-41.

30. Nashan B, Schlitt HJ, Tusch G et al. Biliary malignancies in primary sclerosing cholangitis: timing for liver transplantation. Hepatology 1996;23:1105-11.

31. Broome U, Olsson R, Loof L et al. Natural history and prognostic factors in 305 Swedish patients with primary sclerosing cholangitis. Gut 1996;38:610-5.

32. Kornfeld D, Ekbom A, Ihre T. Survival and risk of cholangiocarcinoma in patients with primary sclerosing cholangitis. A population-based study. Scand J Gastroenterol 1997;32:1042-5.

33. Harnois DM, Gores GJ, Ludwig J, Steers JL, LaRusso NF, Wiesner RH. Are patients with cirrhotic stage primary sclerosing cholangitis at risk for the development of hepatocellular cancer? J Hepatol 1997;27:512-6.

34. Leidenius MH, Farkkila MA, Karkkainen P, Taskinen EI, Kellokumpu IH, Hockerstedt KA. Colorectal dysplasia and carcinoma in patients with ulcerative colitis and primary sclerosing cholangitis. Scand J Gastroenterol 1997;32:706-11.

35. Mendes FD, Kim WR, Pedersen R, Therneau T, Lindor KD. Mortality attributable to cholestatic liver disease in the United States. Hepatology 2008;47:1241-7.

36. Claessen MM, Vleggaar FP, Tytgat KM, Siersema PD, van Buuren HR. High lifetime risk of cancer in primary sclerosing cholangitis. J Hepatol 2009;50:158-64.

37. Rudolph G, Gotthardt D, Kloeters-Plachky P, Rost D, Kulaksiz H, Stiehl A. In PSC with dominant bile duct stenosis, IBD is associated with an increase of carcinomas and reduced survival. J Hepatol 2010;53:313-7.

38. Toy E, Balasubramanian S, Selmi C, Li CS, Bowlus CL. The prevalence, incidence and natural history of primary sclerosing cholangitis in an ethnically diverse population. BMC Gastroenterol 2011;11:83.

39. Dickson ER, Murtaugh PA, Wiesner RH et al. Primary sclerosing cholangitis: refinement and validation of survival models. Gastroenterology 1992;103:1893-901.

40. Olsson RG, Asztely MS. Prognostic value of cholangiography in primary sclerosing cholangitis. Eur J Gastroenterol Hepatol 1995;7:251-4.

41. Ponsioen CY, Vrouenraets SM, Prawirodirdjo W et al. Natural history of primary sclerosing cholangitis and prognostic value of cholangiography in a Dutch population. Gut 2002;51:562-6.

42. Buckles DC, Lindor KD, LaRusso NF, Petrovic LM, Gores GJ. In primary sclerosing cholangitis, gallbladder polyps are frequently malignant. Am J Gastroenterol 2002;97:1138-42.

43. Broome U, Glaumann H, Lindstom E et al. Natural history and outcome in 32 Swedish patients with small duct primary sclerosing cholangitis (PSC). J Hepatol 2002;36:586-9.

44. Feldstein AE, Perrault J, El-Youssif M, Lindor KD, Freese DK, Angulo P. Primary sclerosing cholangitis in children: a long-term follow-up study. Hepatology 2003;38:210-7.

45. Tischendorf JJ, Hecker H, Kruger M, Manns MP, Meier PN. Characterization, outcome, and prognosis in 273 patients with primary sclerosing cholangitis: A single center study. Am J Gastroenterol 2007;102:107-14.

46. Bjornsson E, Olsson R, Bergquist A et al. The natural history of small-duct primary sclerosing cholangitis. Gastroenterology 2008;134:975-80.

47. Said K, Glaumann H, Bergquist A. Gallbladder disease in patients with primary sclerosing cholangitis. J Hepatol 2008;48:598-605.

48. Kornasiewicz O, Lewandowski Z, Dudek K, Stankiewicz R, Nyckowski P, Krawczyk M. Prediction of graft loss and death in patients with primary sclerosing cholangitis. Transplant Proc 2009;41:3110-3.

49. Ataseven H, Parlak E, Yuksel I et al. Primary sclerosing cholangitis in Turkish patients: characteristic features and prognosis. Hepatobiliary Pancreat Dis Int 2009;8:312-5.

50. Kashyap R, Mantry P, Sharma R et al. Comparative analysis of outcomes in living and deceased donor liver transplants for primary sclerosing cholangitis. J Gastrointest Surg 2009;13:1480-6.

51. Garioud A, Seksik P, Chretien Y et al. Characteristics and clinical course of primary sclerosing cholangitis in France: a prospective cohort study. Eur J Gastroenterol Hepatol 2010;22:842-7.

52. Ngu JH, Gearry RB, Wright AJ, Stedman CA. Inflammatory bowel disease is associated with poor outcomes of patients with primary sclerosing cholangitis. Clin Gastroenterol Hepatol 2011;9:1092-7.

53. Lamberts LE, Janse M, Haagsma EB, van den Berg AP, Weersma RK. Immune-mediated diseases in primary sclerosing cholangitis. Dig Liver Dis 2011;43:802-6.

54. Sinakos E, Saenger AK, Keach J, Kim WR, Lindor KD. Many patients with primary sclerosing cholangitis and increased serum levels of carbohydrate antigen 19-9 do not have cholangiocarcinoma. Clin Gastroenterol Hepatol 2011;9:434-9.

55. Chapman MH, Webster GJ, Bannoo S, Johnson GJ, Wittmann J, Pereira SP. Cholangiocarcinoma and dominant strictures in patients with primary sclerosing cholangitis: a 25-year single-centre experience. Eur J Gastroenterol Hepatol 2012;24:1051-8.

56. Hirano K, Tada M, Mizuno S et al. Lower incidence of biliary carcinoma in patients with primary sclerosing cholangitis and high serum levels of immunoglobulin E. Clin Gastroenterol Hepatol 2012;10:79-83.

57. Fevery J, Henckaerts L, Van OR et al. Malignancies and mortality in 200 patients with primary sclerosering cholangitis: a long-term single-centre study. Liver Int 2012;32:214-22.

58. Rupp C, Mummelthei A, Sauer P et al. Non-IBD immunological diseases are a risk factor for reduced survival in PSC. Liver Int 2013;33:86-93.

59. Geramizadeh B, Ghavvas R, Kazemi K, Shamsaeefar A, Nikeghbalian S, Malekhosseini SA. Cholangiocarcinoma Secondary to Primary Sclerosing Cholangitis in Explanted Livers: A Single-Center Study in the South of Iran. Hepat Mon 2015;15:e33626.

60. Eaton JE, Barr Fritcher EG, Gores GJ et al. Biliary multifocal chromosomal polysomy and cholangiocarcinoma in primary sclerosing cholangitis. Am J Gastroenterol 2015;110:299-309.

61. Yanai H, Matalon S, Rosenblatt A et al. Prognosis of primary sclerosing cholangitis in israel is independent of coexisting inflammatory bowel Disease. J Crohns Colitis 2015;9:177-84.

62. Gulamhusein AF, Eaton JE, Tabibian JH, Atkinson EJ, Juran BD, Lazaridis KN. Duration of Inflammatory Bowel Disease Is Associated With Increased Risk of Cholangiocarcinoma in Patients With Primary Sclerosing Cholangitis and IBD. Am J Gastroenterol 2016;111:705-11.

63. Hilscher M, Enders FB, Carey EJ, Lindor KD, Tabibian JH. Alkaline phosphatase normalization is a biomarker of improved survival in primary sclerosing cholangitis. Ann Hepatol 2016;15:246-53.

64. Liu K, Wang R, Kariyawasam V et al. Epidemiology and outcomes of primary sclerosing cholangitis with and without inflammatory bowel disease in an Australian cohort. Liver Int 2017;37:442-8.

65. Post AB, Bozdech JM, Lavery I, Barnes DS. Colectomy in patients with inflammatory bowel disease and primary sclerosing cholangitis. Dis Colon Rectum 1994;37:175-8.

66. Siqueira E, Schoen RE, Silverman W et al. Detecting cholangiocarcinoma in patients with primary sclerosing cholangitis. Gastrointest Endosc 2002;56:40-7.

67. Boberg KM, Rocca G, Egeland T et al. Time-dependent Cox regression model is superior in prediction of prognosis in primary sclerosing cholangitis. Hepatology 2002;35:652-7.

68. Rudolph G, Kloeters-Plachky P, Rost D, Stiehl A. The incidence of cholangiocarcinoma in primary sclerosing cholangitis after long-time treatment with ursodeoxycholic acid. Eur J Gastroenterol Hepatol 2007;19:487-91.

69. Ang TL, Fock KM, Ng TM, Teo EK, Chua TS, Tan JY. Clinical profile of primary sclerosing cholangitis in Singapore. J Gastroenterol Hepatol 2002;17:908-13.

70. Johnson GK, Saeian K, Geenen JE. Primary sclerosing cholangitis treated by endoscopic biliary dilation: review and long-term follow-up evaluation. Curr Gastroenterol Rep 2006;8:147-55.

71. Morris-Stiff G, Bhati C, Olliff S et al. Cholangiocarcinoma complicating primary sclerosing cholangitis: a 24-year experience. Dig Surg 2008;25:126-32.

72. Kaya M, de Groen PC, Angulo P et al. Treatment of cholangiocarcinoma complicating primary sclerosing cholangitis: the Mayo Clinic experience. Am J Gastroenterol 2001;96:1164-9.

73. Chalasani N, Baluyut A, Ismail A et al. Cholangiocarcinoma in patients with primary sclerosing cholangitis: a multicenter case-control study. Hepatology 2000;31:7-11.

74. Pardi DS, Loftus EV, Jr., Kremers WK, Keach J, Lindor KD. Ursodeoxycholic acid as a chemopreventive agent in patients with ulcerative colitis and primary sclerosing cholangitis. Gastroenterology 2003;124:889-93.

75. Olsson R, Boberg KM, de Muckadell OS et al. High-dose ursodeoxycholic acid in primary sclerosing cholangitis: a 5-year multicenter, randomized, controlled study. Gastroenterology 2005;129:1464-72.

76. Wolf JM, Rybicki LA, Lashner BA. The impact of ursodeoxycholic acid on cancer, dysplasia and mortality in ulcerative colitis patients with primary sclerosing cholangitis. Aliment Pharmacol Ther 2005;22:783-8.

77. Zhou YM, Yin ZF, Yang JM et al. Risk factors for intrahepatic cholangiocarcinoma: a case-control study in China. World J Gastroenterol 2008;14:632-5.

78. Melum E, Karlsen TH, Schrumpf E et al. Cholangiocarcinoma in primary sclerosing cholangitis is associated with NKG2D polymorphisms. Hepatology 2008;47:90-6.

79. Tanaka M, Tanaka H, Tsukuma H, Ioka A, Oshima A, Nakahara T. Risk factors for intrahepatic cholangiocarcinoma: a possible role of hepatitis B virus. J Viral Hepat 2010;17:742-8.

80. Brentnall TA, Haggitt RC, Rabinovitch PS et al. Risk and natural history of colonic neoplasia in patients with primary sclerosing cholangitis and ulcerative colitis. Gastroenterology 1996;110:331-8.

81. Graziadei IW, Wiesner RH, Marotta PJ et al. Long-term results of patients undergoing liver transplantation for primary sclerosing cholangitis. Hepatology 1999;30:1121-7.

82. Maheshwari A, Yoo HY, Thuluvath PJ. Long-term outcome of liver transplantation in patients with PSC: a comparative analysis with PBC. Am J Gastroenterol 2004;99:538-42.

83. Parlak E, Ulker A, Alkim C et al. The course of colonic disease in ulcerative colitis patients with primary sclerosing cholangitis. Turk J Gastroenterol 2002;13:134-8.

84. Loftus EV, Jr., Harewood GC, Loftus CG et al. PSC-IBD: a unique form of inflammatory bowel disease associated with primary sclerosing cholangitis. Gut 2005;54:91-6.

85. van den Brand FF, van d, V, de Boer YS et al. Increased Mortality Among Patients With vs Without Cirrhosis and Autoimmune Hepatitis. Clinical Gastroenterology and Hepatology 2019;17:940-7.

86. Lindstrom L, Lapidus A, Ost A, Bergquist A. Increased risk of colorectal cancer and dysplasia in patients with Crohn's colitis and primary sclerosing cholangitis. Dis Colon Rectum 2011;54:1392-7.

87. Sokol H, Cosnes J, Chazouilleres O et al. Disease activity and cancer risk in inflammatory bowel disease associated with primary sclerosing cholangitis. World J Gastroenterol 2008;14:3497-503.

88. Claessen MM, Lutgens MW, van Buuren HR et al. More right-sided IBD-associated colorectal cancer in patients with primary sclerosing cholangitis. Inflamm Bowel Dis 2009;15:1331-6.

89. Navaneethan U, Choure A, Venkatesh PG et al. Presence of concomitant inflammatory bowel disease is associated with an increased risk of postcholecystectomy complications. Inflamm Bowel Dis 2012;18:1682-8.

90. Braden B, Halliday J, Aryasingha S et al. Risk for colorectal neoplasia in patients with colonic Crohn's disease and concomitant primary sclerosing cholangitis. Clin Gastroenterol Hepatol 2012;10:303-8.

91. Baars JE, Looman CW, Steyerberg EW et al. The risk of inflammatory bowel disease-related colorectal carcinoma is limited: results from a nationwide nested case-control study. Am J Gastroenterol 2011;106:319-28.

92. Hanouneh IA, Macaron C, Lopez R, Zein NN, Lashner BA. Risk of colonic neoplasia after liver transplantation for primary sclerosing cholangitis. Inflamm Bowel Dis 2012;18:269-74.

93. Vera A, Gunson BK, Ussatoff V et al. Colorectal cancer in patients with inflammatory bowel disease after liver transplantation for primary sclerosing cholangitis. Transplantation 2003;75:1983-8.

94. Fraga M, Fournier N, Safroneeva E et al. Primary sclerosing cholangitis in the Swiss Inflammatory Bowel Disease Cohort Study: prevalence, risk factors, and long-term follow-up. Eur J Gastroenterol Hepatol 2017;29:91-7.

95. Jess T, Loftus EV, Jr., Velayos FS et al. Risk factors for colorectal neoplasia in inflammatory bowel disease: a nested case-control study from Copenhagen county, Denmark and Olmsted county, Minnesota. Am J Gastroenterol 2007;102:829-36.

96. Ananthakrishnan AN, Cagan A, Gainer VS et al. Mortality and extraintestinal cancers in patients with primary sclerosing cholangitis and inflammatory bowel disease. J Crohns Colitis 2014;8:956-63.

97. Manninen P, Karvonen AL, Laukkarinen J, Aitola P, Huhtala H, Collin P. Colorectal cancer and cholangiocarcinoma in patients with primary sclerosing cholangitis and inflammatory bowel disease. Scand J Gastroenterol 2015;50:423-8.

98. Brandsaeter B, Friman S, Broome U et al. Outcome following liver transplantation for primary sclerosing cholangitis in the Nordic countries. Scand J Gastroenterol 2003;38:1176-83.

99. Sint NJ, Tjon AS, Metselaar HJ, Kuipers EJ, de Man RA, van Leerdam ME. Colorectal cancer in post-liver transplant recipients. Dis Colon Rectum 2010;53:817-21.

100. Rompianesi G, Ravikumar R, Jose S et al. Incidence and outcome of colorectal cancer in liver transplant recipients: A national, multicentre analysis on 8115 patients. Liver International 2019;39:353-60.

101. Fausa O, Kolmannskog F, Ritland S. The pancreatic ducts in primary biliary cirrhosis and sclerosing cholangitis. Scand J Gastroenterol Suppl 1985;107:32-5.

102. Shetty K, Rybicki L, Brzezinski A, Carey WD, Lashner BA. The risk for cancer or dysplasia in ulcerative colitis patients with primary sclerosing cholangitis. Am J Gastroenterol 1999;94:1643-9.

103. Kornfeld D, Ekbom A, Ihre T. Is there an excess risk for colorectal cancer in patients with ulcerative colitis and concomitant primary sclerosing cholangitis? A population based study. Gut 1997;41:522-5.

104. Marchesa P, Lashner BA, Lavery IC et al. The risk of cancer and dysplasia among ulcerative colitis patients with primary sclerosing cholangitis. Am J Gastroenterol 1997;92:1285-8.

105. Lakatos L, Mester G, Erdelyi Z et al. Risk factors for ulcerative colitis-associated colorectal cancer in a Hungarian cohort of patients with ulcerative colitis: results of a population-based study. Inflamm Bowel Dis 2006;12:205-11.

106. Terg R, Sambuelli A, Coronel E et al. Prevalence of primary sclerosing cholangitis in patients with ulcerative colitis and the risk of developing malignancies. A large prospective study. Acta Gastroenterol Latinoam 2008;38:26-33.

107. D'Haens GR, Lashner BA, Hanauer SB. Pericholangitis and sclerosing cholangitis are risk factors for dysplasia and cancer in ulcerative colitis. Am J Gastroenterol 1993;88:1174-8.

108. Nuako KW, Ahlquist DA, Sandborn WJ, Mahoney DW, Siems DM, Zinsmeister AR. Primary sclerosing cholangitis and colorectal carcinoma in patients with chronic ulcerative colitis: a case-control study. Cancer 1998;82:822-6.

109. Navaneethan U, Kochhar G, Venkatesh PG et al. Duration and severity of primary sclerosing cholangitis is not associated with risk of neoplastic changes in the colon in patients with ulcerative colitis. Gastrointest Endosc 2012;75:1045-54.

110. Navaneethan U, Venkatesh PG, Lashner BA, Remzi FH, Shen B, Kiran RP. Temporal trends in colon neoplasms in patients with primary sclerosing cholangitis and ulcerative colitis. J Crohns Colitis 2012;6:845-51.

111. Loftus EV, Jr., Aguilar HI, Sandborn WJ et al. Risk of colorectal neoplasia in patients with primary sclerosing cholangitis and ulcerative colitis following orthotopic liver transplantation. Hepatology 1998;27:685-90.

112. Navaneethan U, Venkatesh PG, Lashner BA, Shen B, Kiran RP. The impact of ulcerative colitis on the long-term outcome of patients with primary sclerosing cholangitis. Aliment Pharmacol Ther 2012;35:1045-53.

113. Marsh JW, Jr., Iwatsuki S, Makowka L et al. Orthotopic liver transplantation for primary sclerosing cholangitis. Ann Surg 1988;207:21-5.

114. Schrumpf E, Abdelnoor M, Fausa O, Elgjo K, Jenssen E, Kolmannskog F. Risk factors in primary sclerosing cholangitis. J Hepatol 1994;21:1061-6.

115. Van Laethem JL, Deviere J, Bourgeois N et al. Cholangiographic findings in deteriorating primary sclerosing cholangitis. Endoscopy 1995;27:223-8.

116. Ahrendt SA, Pitt HA, Kalloo AN et al. Primary sclerosing cholangitis: resect, dilate, or transplant? Ann Surg 1998;227:412-23.

117. Kim WR, Therneau TM, Wiesner RH et al. A revised natural history model for primary sclerosing cholangitis. Mayo Clin Proc 2000;75:688-94.

118. Boberg KM, Bergquist A, Mitchell S et al. Cholangiocarcinoma in primary sclerosing cholangitis: risk factors and clinical presentation. Scand J Gastroenterol 2002;37:1205-11.

119. Goet JC, Floreani A, Verhelst X et al. Validation, clinical utility and limitations of the Amsterdam-Oxford model for primary sclerosing cholangitis. Journal of Hepatology 71 (5) (pp 992-999), 2019;71:992-9.

120. Guerra I, Bujanda L, Castro J et al. Clinical Characteristics, Associated Malignancies and Management of Primary Sclerosing Cholangitis in Inflammatory Bowel Disease Patients: A Multicentre Retrospective Cohort Study. Journal of Crohn's & colitis 2019;13:1492-500.

121. Bhat M, Mara K, DIerkhising R, Watt KD. Gender, Race and Disease Etiology Predict de Novo Malignancy Risk after Liver Transplantation: Insights for Future Individualized Cancer Screening Guidance. Transplantation 2019;103:91-100.

122. Kuo A, Gomel R, Safer R, Lindor KD, Everson GT, Bowlus CL. Characteristics and Outcomes Reported by Patients With Primary Sclerosing Cholangitis Through an Online Registry. Clinical Gastroenterology and Hepatology 2019;17:1372-8.

123. Freeman E, Majeed A, Kemp W, Roberts SK. Long-term outcomes of primary sclerosing cholangitis: an Australian non-transplant tertiary hospital perspective. Internal Medicine Journal 2019;49:323-7.

124. Aadland E, Schrumpf E, Fausa O et al. Primary sclerosing cholangitis: a long-term follow-up study. Scand J Gastroenterol 1987;22:655-64.

125. Saldeen K, Friman S, Olausson M, Olsson R. Follow-up after liver transplantation for primary sclerosing cholangitis: effects on survival, quality of life, and colitis. Scand J Gastroenterol 1999;34:535-40.

126. Zenouzi R, Weismuller TJ, Hubener P et al. Low risk of hepatocellular carcinoma in patients with primary sclerosing cholangitis with cirrhosis. Clin Gastroenterol Hepatol 2014;12:1733-8.

127. Weismuller TJ, Gulamhusein A, Trivedi P et al. Impact of geographical region on death or transplantation and on malignancy in primary sclerosing cholangitis-a multicenter retrospective study of the international PSC study group (IPSCSG). Hepatology Conference: 69th Annual Meeting of the American Association for the Study of Liver Diseases, AASLD 2018;October.

128. van EL, Cunningham M, Narasimman M et al. Risk of gall bladder cancer in patients with primary sclerosing cholangitis and gall bladder polyps: an opportunity to revisit the guidelines. Journal of Hepatology Conference: The International Liver Congress 2019;2019.

129. Bambha K, Kim WR, Talwalkar J et al. Incidence, clinical spectrum, and outcomes of primary sclerosing cholangitis in a United States community. Gastroenterology 2003;125:1364-9.

130. Farrant JM, Hayllar KM, Wilkinson ML et al. Natural history and prognostic variables in primary sclerosing cholangitis. Gastroenterology 1991;100:1710-7.

131. van Erp LW, Cunningham M, Narasimman M et al. Risk of gallbladder cancer in patients with primary sclerosing cholangitis and radiographically detected gallbladder polyps. Liver International 2020;40:382-92.

132. Milkiewicz P, Mutimer D, Hubscher SG, Elias E. Autoimmune liver disease in patients with neoplastic diseases. Eur J Gastroenterol Hepatol 1999;11:569-73.

133. Mir-Madjlessi SH, Farmer RG, Sivak MV, Jr. Bile duct carcinoma in patients with ulcerative colitis. Relationship to sclerosing cholangitis: report of six cases and review of the literature. Dig Dis Sci 1987;32:145-54.

134. Porayko MK, LaRusso NF, Wiesner RH. Primary sclerosing cholangitis: a progressive disease? Semin Liver Dis 1991;11:18-25.

135. Rosen CB, Nagorney DM. Cholangiocarcinoma complicating primary sclerosing cholangitis. Semin Liver Dis 1991;11:26-30.

136. Rosen CB, Nagorney DM, Wiesner RH, Coffey RJ, Jr., LaRusso NF. Cholangiocarcinoma complicating primary sclerosing cholangitis. Ann Surg 1991;213:21-5.

137. Wiesner RH, Porayko MK, Hay JE et al. Liver transplantation for primary sclerosing cholangitis: impact of risk factors on outcome. Liver Transpl Surg 1996;2:99-108.

138. Thuluvath PJ, Rai R, Venbrux AC, Yeo CJ. Cholangiocarcinoma: a review. Gastroenterologist 1997;5:306-15.

139. Wiesner RH. Liver transplantation for primary biliary cirrhosis and primary sclerosing cholangitis: predicting outcomes with natural history models. Mayo Clin Proc 1998;73:575-88.

140. Narayanan Menon KV, Wiesner RH. Etiology and natural history of primary sclerosing cholangitis. J Hepatobiliary Pancreat Surg 1999;6:343-51.

141. Chapman RW. Risk factors for biliary tract carcinogenesis. Ann Oncol 1999;10 Suppl 4:308-11.

142. Sherlock S. Primary biliary cirrhosis, primary sclerosing cholangitis, and autoimmune cholangitis. Clin Liver Dis 2000;4:97-113.

143. Prall RT, Lindor KD, Wiesner RH, LaRusso NF. Current therapies and clinical controversies in the management of primary sclerosing cholangitis. Curr Gastroenterol Rep 2000;2:99-103.

144. Stiehl A, Benz C, Sauer P. Primary sclerosing cholangitis. Can J Gastroenterol 2000;14:311-5.

145. Tranter SE, Maddern GJ, Berry DP. Inflammatory conditions of the common bile duct. Aust N Z J Surg 2000;70:95-7.

146. Talwalkar JA, Lindor KD. Natural history and prognostic models in primary sclerosing cholangitis. Best Pract Res Clin Gastroenterol 2001;15:563-75.

147. Ahrendt SA, Nakeeb A, Pitt HA. Cholangiocarcinoma. Clin Liver Dis 2001;5:191-218.

148. Jayaram H, Satsangi J, Chapman RW. Increased colorectal neoplasia in chronic ulcerative colitis complicated by primary sclerosing cholangitis: fact or fiction? Gut 2001;48:430-4.

149. Lashner BA. Colorectal cancer surveillance for patients with inflammatory bowel disease. Gastrointest Endosc Clin N Am 2002;12:135-43, viii.

150. MacFaul GR, Chapman RW. Sclerosing cholangitis. Curr Opin Gastroenterol 2004;20:275-80.

151. Shaib Y, El-Serag HB. The epidemiology of cholangiocarcinoma. Semin Liver Dis 2004;24:115-25.

152. MacFaul GR, Chapman RW. Sclerosing cholangitis. Curr Opin Gastroenterol 2005;21:348-53.

153. Talwalkar JA, Lindor KD. Primary sclerosing cholangitis. Inflamm Bowel Dis 2005;11:62-72.

154. Worthington J, Chapman R. Primary sclerosing cholangitis. Orphanet J Rare Dis 2006;1:41.

155. LaRusso NF, Shneider BL, Black D et al. Primary sclerosing cholangitis: summary of a workshop. Hepatology 2006;44:746-64.

156. MacFaul GR, Chapman RW. Sclerosing cholangitis. Curr Opin Gastroenterol 2006;22:288-93.

157. Lazaridis KN, Gores GJ. Primary sclerosing cholangitis and cholangiocarcinoma. Semin Liver Dis 2006;26:42-51.

158. Broome U, Bergquist A. Primary sclerosing cholangitis, inflammatory bowel disease, and colon cancer. Semin Liver Dis 2006;26:31-41.

159. Levy C, Lindor KD. Primary sclerosing cholangitis: epidemiology, natural history, and prognosis. Semin Liver Dis 2006;26:22-30.

160. Ben-Menachem T. Risk factors for cholangiocarcinoma. Eur J Gastroenterol Hepatol 2007;19:615-7.

161. Geonzon-Gonzales MR. Primary sclerosing cholangitis. Gastroenterol Nurs 2007;30:102-5.

162. Fevery J, Verslype C, Lai G, Aerts R, Van SW. Incidence, diagnosis, and therapy of cholangiocarcinoma in patients with primary sclerosing cholangitis. Dig Dis Sci 2007;52:3123-35.

163. Maggs JR, Chapman RW. Sclerosing cholangitis. Curr Opin Gastroenterol 2007;23:310-6.

164. Ustundag Y, Bayraktar Y. Cholangiocarcinoma: a compact review of the literature. World J Gastroenterol 2008;14:6458-66.

165. Khan SA, Toledano MB, Taylor-Robinson SD. Epidemiology, risk factors, and pathogenesis of cholangiocarcinoma. HPB (Oxford) 2008;10:77-82.

166. Silveira MG, Lindor KD. Primary sclerosing cholangitis. Can J Gastroenterol 2008;22:689-98.

167. Kitiyakara T, Chapman RW. Chemoprevention and screening in primary sclerosing cholangitis. Postgrad Med J 2008;84:228-37.

168. Weismuller TJ, Wedemeyer J, Kubicka S, Strassburg CP, Manns MP. The challenges in primary sclerosing cholangitis--aetiopathogenesis, autoimmunity, management and malignancy. J Hepatol 2008;48 Suppl 1:S38-S57.

169. Yachimski P, Pratt DS. Cholangiocarcinoma: natural history, treatment, and strategies for surveillance in high-risk patients. J Clin Gastroenterol 2008;42:178-90.

170. Schulick RD. Primary sclerosing cholangitis: detection of cancer in strictures. J Gastrointest Surg 2008;12:420-2.

171. Abbas G, Lindor KD. Cholangiocarcinoma in primary sclerosing cholangitis. J Gastrointest Cancer 2009;40:19-25.

172. Jesudian AB, Jacobson IM. Screening and diagnosis of cholangiocarcinoma in patients with primary sclerosing cholangitis. Rev Gastroenterol Disord 2009;9:E41-E47.

173. Karlsen TH, Schrumpf E, Boberg KM. Primary sclerosing cholangitis. Best Pract Res Clin Gastroenterol 2010;24:655-66.

174. Mendes F, Lindor KD. Primary sclerosing cholangitis: overview and update. Nat Rev Gastroenterol Hepatol 2010;7:611-9.

175. Krejs GJ. Pancreatic cancer: epidemiology and risk factors. Dig Dis 2010;28:355-8.

176. Gatto M, Alvaro D. Cholangiocarcinoma: risk factors and clinical presentation. Eur Rev Med Pharmacol Sci 2010;14:363-7.

177. Razumilava N, Gores GJ, Lindor KD. Cancer surveillance in patients with primary sclerosing cholangitis. Hepatology 2011;54:1842-52.

178. Torres J, Pineton de CG, Itzkowitz S, Sachar DB, Colombel JF. Review article: colorectal neoplasia in patients with primary sclerosing cholangitis and inflammatory bowel disease. Aliment Pharmacol Ther 2011;34:497-508.

179. Tyson GL, El-Serag HB. Risk factors for cholangiocarcinoma. Hepatology 2011;54:173-84.

180. Basseri RJ, Basseri B, Papadakis KA. Dysplasia and cancer in inflammatory bowel disease. Expert Rev Gastroenterol Hepatol 2011;5:59-66.

181. Andersen V, Halfvarson J, Vogel U. Colorectal cancer in patients with inflammatory bowel disease: can we predict risk? World J Gastroenterol 2012;18:4091-4.

182. Dyson JK, Rutter MD. Colorectal cancer in inflammatory bowel disease: what is the real magnitude of the risk? World J Gastroenterol 2012;18:3839-48.

183. Rustagi T, Dasanu CA. Risk factors for gallbladder cancer and cholangiocarcinoma: similarities, differences and updates. J Gastrointest Cancer 2012;43:137-47.

184. Ehlken H, Schramm C. Primary sclerosing cholangitis and cholangiocarcinoma: pathogenesis and modes of diagnostics. Dig Dis 2013;31:118-25.

185. Zein CO. Primary sclerosing cholangitis. Clin Liver Dis 2013;17:211-27.

186. Wang R, Leong RW. Primary sclerosing cholangitis as an independent risk factor for colorectal cancer in the context of inflammatory bowel disease: a review of the literature. World J Gastroenterol 2014;20:8783-9.

187. Bonato G, Cristoferi L, Strazzabosco M, Fabris L. Malignancies in Primary Sclerosing Cholangitis--A Continuing Threat. Dig Dis 2015;33 Suppl 2:140-8.

188. Ehlken H, Schramm C. How Should Cancer Surveillance in Primary Sclerosing Cholangitis Be Performed? Viszeralmedizin 2015;31:173-7.

189. Rizvi S, Eaton JE, Gores GJ. Primary Sclerosing Cholangitis as a Premalignant Biliary Tract Disease: Surveillance and Management. Clin Gastroenterol Hepatol 2015;13:2152-65.

190. Schrumpf E, Boberg KM, Karlsen TH. Primary sclerosing cholangitis - the Norwegian experience. Scand J Gastroenterol 2015;50:781-96.

191. Khaderi SA, Sussman NL. Screening for malignancy in primary sclerosing cholangitis (PSC). Curr Gastroenterol Rep 2015;17:17.

192. Folseraas T, Boberg KM. Cancer Risk and Surveillance in Primary Sclerosing Cholangitis. Clin Liver Dis 2016;20:79-98.

193. Ehlken H, Zenouzi R, Schramm C. Risk of cholangiocarcinoma in patients with primary sclerosing cholangitis: diagnosis and surveillance. Curr Opin Gastroenterol 2017;33:78-84.

194. Rao BB, Lashner B, Kowdley KV. Reviewing the Risk of Colorectal Cancer in Inflammatory Bowel Disease after Liver Transplantation for Primary Sclerosing Cholangitis. Inflammatory Bowel Diseases 24 (2) (pp 269-276), 2018;18.

195. Fung BM, Tabibian JH. Cholangiocarcinoma in patients with primary sclerosing cholangitis. Curr Opin Gastroenterol 2019.

196. Song J, Li Y, Bowlus CL, Yang G, Leung PSC, Gershwin ME. Cholangiocarcinoma in Patients with Primary Sclerosing Cholangitis (PSC): a Comprehensive Review. Clin Rev Allergy Immunol 2019.

197. Hamaoka M, Kozaka K, Matsui O et al. Early detection of intrahepatic cholangiocarcinoma. Jpn J Radiol 2019;37:669-84.

198. Lleo A, de Boer YS, Liberal R, Colombo M. The risk of liver cancer in autoimmune liver diseases. Ther Adv Med Oncol 2019;11:1758835919861914.

199. Chascsa DM, Lindor KD. Cancer risk, screening and surveillance in primary sclerosing cholangitis. Minerva Gastroenterol Dietol 2019;65:214-28.

200. Fricker ZP, Lichtenstein DR. Primary Sclerosing Cholangitis: A Concise Review of Diagnosis and Management. Dig Dis Sci 2019;64:632-42.

201. Bowlus CL, Lim JK, Lindor KD. AGA Clinical Practice Update on Surveillance for Hepatobiliary Cancers in Patients With Primary Sclerosing Cholangitis: Expert Review. Clinical Gastroenterology and Hepatology 2019;17:2416-22.

202. Salazar M, Ituarte C, Abriata MG, Santoro F, Arroyo G. Gallbladder cancer in South America: Epidemiology and prevention. Chinese Clinical Oncology 2019;8:32.

203. Kim H-S, El-Serag HB. The Epidemiology of Hepatocellular Carcinoma in the USA. Current Gastroenterology Reports 2019;21:17.

204. Thomas T, Cooney R, Iqbal T et al. Colorectal cancer, colectomy rates and inflammatory bowel disease activity following liver transplantation in primary sclerosing cholangitis: A systematic review and meta-analysis. Journal of Hepatology Conference: The International Liver Congress 2019;April.

205. Thomas T, Cooney R, Iqbal T et al. Colorectal cancer, colectomy rates and inflammatory bowel disease activity following liver transplantation in primary sclerosing cholangitis: A systematic review and meta-analysis. Journal of Crohn's and Colitis Conference: 14th Annual Congress of the European Crohn's and Colitis Organisation, ECCO 2019;March.

206. Khan SA, Tavolari S, Brandi G. Cholangiocarcinoma: Epidemiology and risk factors. Liver International 2019;39:19-31.

207. Khan AS, Dageforde LA. Cholangiocarcinoma. Surgical Clinics of North America 2019;99:315-35.

208. Fung BM, Lindor KD, Tabibian JH. Cancer risk in primary sclerosing cholangitis: Epidemiology, prevention, and surveillance strategies. World Journal of Gastroenterology 2019;25:659-71.

209. Thomas T, Cooney R, Iqbal T et al. Colorectal cancer, colectomy and inflammatory bowel disease activity following liver transplantation in primary sclerosing cholangitis. Gut Conference: Annual Meeting of the British Society of Gastroenterology, BSG 2019;June.

210. Sint NJ, de J, V, Steyerberg EW, Kuipers EJ, van Leerdam ME, Veldhuyzen-van Zanten SJ. Risk of colorectal carcinoma in post-liver transplant patients: a systematic review and meta-analysis. Am J Transplant 2010;10:868-76.

Supplementary Table 2. Prospective studies of primary sclerosing cholangitis and cholangiocarcinoma

First author, publication year, country	Study name	Study period	Number of participants, number of cases	Exposure, subgroup	Comparison	Relative risk (95% confidence interval)	Adjustment for confounders
Burak K et al, 2004, USA	Mayo Clinic Rochester	1970-1984 - 1997, 11.5 years follow-up years follow-up	161 PSC patients, men and women, mean age 40.7 years: 11 cholangiocarcinoma cases	PSC	Yes vs. no	1560 (780-2793)	Age, sex
de Valle MB et al, 2012, Sweden	Vastra Gotaland	1992-2005 - 2008, 6.5 years follow-up	199 PSC patients, men and women, age 18.2-76.8 years: 17 cholangiocarcinoma cases	PSC	Yes vs. no	868 (505-1390)	Age, sex
Boonstra K et al, 2013, Netherlands	Groningen, Rotterdam, Leiden	2000-2007 - 2012, 7.7 years follow-up	590 PSC patients, men and women, mean age 38.9 years: 44 cholangiocarcinoma cases	PSC	Yes vs. no	398 (246-608)	Age, sex
Barner-Rasmussen N et al, 2020, Finland	Hospital District of Helsinki and Uusimaa (HUS)	1990-2015, ~25 years follow-up	632 PSC patients, mean age 40.6 years: 20 cholangiocarcinoma cases<br>15 cholangiocarcinoma deaths	PSC, incidence<br>PSC, mortality	Yes vs. no<br>Yes vs. no	235 (143-362)<br>244 (137-402)	Age, sex, year

PSC: primary sclerosing cholangitis

Supplementary Table 3. Prospective studies of primary sclerosing cholangitis and hepatobiliary cancer

First author, publication year, country	Study name	Study period	Number of participants, number of cases	Exposure, subgroup	Comparison	Relative risk (95% confidence interval)	Adjustment for confounders
Bergquist A et al, 2002, Sweden	Swedish Cancer and Death registries	1970-1998 - 1998, 5.7 years follow-up	604 PSC patients, men and women, mean age 40.7 years: 53 hepatobiliary cancer cases (31 cases excluding 1st year)	PSC<br>PSC, excluding 1st year of follow-up	Yes vs. no<br>Yes vs. no	160.6 (120.3-210.1)<br>106.9 (72.6-151.7)	Age, sex, calendar-time
de Valle MB et al, 2012, Sweden	Vastra Gotaland	1992-2005 - 2008, 6.5 years follow-up	199 PSC patients, men and women, age 18.2-76.8 years: 21 hepatobiliary tract cancer cases	PSC<br>PSC, excluding cases diagnosed within 1 year after PSC diagnosis	Yes vs. no<br>Yes vs. no	177 (110-271)<br>118 (64.8-199)	Age, sex
Ngu JH et al, 2012, New Zealand	Canterbury	1980-2008 - 2010, 7 years follow-up	81 PSC patients, men and women, age 17-82 years: NA cancer deaths<br>11 hepatobiliary cancer cases	PSC, hepatobiliary cancer mortality<br>PSC, extrahepatic cancer mortality<br>PSC, hepatobiliary cancer incidence	Yes vs. no<br>Yes vs. no<br>Yes vs. no	116.9 (66.8-189.8)<br>1.5 (0.3-4.3)<br>105.2 (50.5-193.5)	Age, sex

NA: not available, PSC: primary sclerosing cholangitis

Supplementary Table 4. Prospective studies of primary sclerosing cholangitis and liver cancer

First author, publication year, country	Study name	Study period	Number of participants, number of cases	Exposure, subgroup	Comparison	Relative risk (95% confidence interval)	Adjustment for confounders
Card TR et al, 2008, United Kingdom	UK General Practice Research Database	1991-2001 - 2001, ~3.1 years follow-up	223 PSC patients and 2217 controls, men and women, age 0-≥85 years: 14 liver cancer cases	PSC	Yes vs. no	41.52 (11.43-150.80)	Age, sex, smoking status
Barner-Rasmussen N et al, 2020, Finland	Hospital District of Helsinki and Uusimaa (HUS)	1990-2015, ~25 years follow-up	632 PSC patients, mean age 40.6 years: 3 hepatocellular carcinoma cases<br>2 hepatocellular carcinoma deaths	PSC, incidence<br>PSC, mortality	Yes vs. no<br>Yes vs. no	21.6 (4.45-63.1)<br>22.1 (2.67-79.7)	Age, sex, year

PSC: primary sclerosing cholangitis

Supplementary Table 5. Prospective studies of primary sclerosing cholangitis and gastrointestinal cancer

First author, publication year, country	Study name	Study period	Number of participants, number of cases	Exposure, subgroup	Comparison	Relative risk (95% confidence interval)	Adjustment for confounders
Bergquist A et al, 2002, Sweden	Swedish Cancer and Death registries	1970-1998 - 1998, 5.7 years follow-up	604 PSC patients, men and women, mean age 40.7 years: 71 gastrointestinal cancer cases (esophagus, stomach, small intestine, CRC, hepatobiliary, pancreas)	PSC	Yes vs. no	28.6 (22.4-36.1)	Age, sex, calendar-time
Card TR et al, 2008, United Kingdom	UK General Practice Research Database	1991-2001 - 2001, ~3.1 years follow-up	223 PSC patients and 2217 controls, men and women, age 0-≥85 years: 27 gastrointestinal cancer cases (including pancreas, but not further defined)	PSC	Yes vs. no	2.53 (0.95-6.74)	Age, sex, smoking status
de Valle MB et al, 2012, Sweden	Vastra Gotaland	1992-2005 - 2008, 6.5 years follow-up	199 PSC patients, men and women, age 18.2-76.8 years: 24 gastrointestinal cancer cases (liver, bile ducts, gallbladder, abdominal carcinomatosis, CRC)	PSC	Yes vs. no	19.3 (12.3-28.7)	Age, sex
Søgaard KK et al, 2014, Denmark	Danish medical registries	1994-2010 - 2010, 3.1 years follow-up	178 PSC patients, men and women, age <65 and ≥65 years: 15 gastrointestinal cancer cases (esophagus, stomach, small intestine, CRC, anal, liver, gallbladder, biliary tract, pancreas)	PSC<br>PSC, 0-<6 months follow-up<br>PSC, 6-<12<br>PSC, 1-<5 years<br>PSC, ≥5 years	Yes vs. no	31.5 (17.6-51.9)<br>114 (52.0-216)<br>31.9 (3.86-115)<br>11.0 (2.26-32.1)<br>16.2 (0.41-90.1)	Age, sex, calendar year

CRC: colorectal cancer, PSC: primary sclerosing cholangitis

Supplementary Table 6. Prospective studies of primary sclerosing cholangitis and pancreatic cancer

First author, publication year, country	Study name	Study period	Number of participants, number of cases	Exposure, subgroup	Comparison	Relative risk (95% confidence interval)	Adjustment for confounders
Bergquist A et al, 2002, Sweden	Swedish Cancer and Death registries	1970-1998 - 1998, 5.7 years follow-up	604 PSC patients, men and women, mean age 40.7 years: 5 pancreatic cancer cases (3 cases excluding 1st year)	PSC<br>PSC, excluding 1st year of follow-up	Yes vs. no<br>Yes vs. no	14.3 (4.7-33.4)<br>9.7 (2.0-18.4)	Age, sex, calendar-time
Liang H et al, 2017, United Kingdom	UK Clinical Practice Research Datalink (CPRD)	1998-2014 - 2014, 5 years follow-up	250 PSC patients and 1250 controls, men and women, age 6-93 years: <9 pancreatic cancer cases	PSC	Yes vs. no	2.93 (0.05-56.2)	Age, sex
Barner-Rasmussen N et al, 2020, Finland	Hospital District of Helsinki and Uusimaa (HUS)	1990-2015, ~25 years follow-up	632 PSC patients, mean age 40.6 years: 2 pancreatic cancer cases 3 pancreatic cancer deaths	PSC, incidence<br>PSC, mortality	Yes vs. no<br>Yes vs. no	3.06 (0.37-11.0)<br>5.53 (1.14-16.2)	Age, sex, year

PSC: primary sclerosing cholangitis

Supplementary Table 7. Prospective studies of primary sclerosing cholangitis and colorectal cancer

First author, publication year, country	Study name	Study period	Number of participants, number of cases	Exposure, subgroup	Comparison	Relative risk (95% confidence interval)	Adjustment for confounders
Loftus EV et al, 1996, USA	Mayo Clinic Minnesota	1984-1988 - 1988, 4.8 years follow-up	178 PSC patients, men and women, mean age 40.3 years: 4 CRC cases	PSC and UC<br>PSC alone	Yes vs. no<br>Yes vs. no	10.3 (2.1-30)<br>4.9 (0.1-27)	Age, sex
Bergquist A et al, 2002, Sweden	Swedish Cancer and Death registries	1970-1998 - 1998, 5.7 years follow-up	604 PSC patients, men and women, mean age 40.7 years: 12 CRC cases (7 cases excluding 1st year)	PSC<br>PSC, excluding 1st year of follow-up	Yes vs. no<br>Yes vs. no	10.3 (5.3-18.1)<br>6.8 (2.7-14.0)	Age, sex, calendar-time
de Valle MB et al, 2012, Sweden	Vastra Gotaland	1992-2005 - 2008, 6.5 years follow-up	199 PSC patients, men and women, age 18.2-76.8 years: 2 CRC cases	PSC	Yes vs. no	2.87 (0.33-10.4)	Age, sex
Ngu JH et al, 2012, New Zealand	Canterbury	1980-2008 - 2010, 7 years follow-up	81 PSC patients, men and women, age 17-82 years: 5 CRC cases	PSC	Yes vs. no	7.0 (2.3-16.3)	Age, sex
Boonstra K et al, 2013, Netherlands	Groningen, Rotterdam, Leiden	2000-2007 - 2012, 7.7 years follow-up	590 PSC patients, men and women, mean age 38.9 years: 20 CRC cases	PSC	Yes vs. no	5.0 (2.02-10.3)	Age, sex
Liang H et al, 2017, United Kingdom	UK Clinical Practice Research Datalink (CPRD)	1998-2014 - 2014, 5 years follow-up	246 PSC patients and 1244 controls, men and women, age 6-93 years: 20 CRC cases	PSC	Yes vs. no	2.5 (0.8-7.0)	Age, sex
Barner-Rasmussen N et al, 2020, Finland	Hospital District of Helsinki and Uusimaa (HUS)	1990-2015, ~25 years follow-up	632 PSC patients, mean age 40.6 years: 5 colon cancer cases<br>3 RC cases<br>1 CC death<br>1 RC death	PSC, CC incidence<br>PSC, RC incidence<br>PSC, CC mortality<br>PSC, RC mortality	Yes vs. no<br>Yes vs. no<br>Yes vs. no<br>Yes vs. no	5.23 (1.70-12.2)<br>5.04 (1.04-14.7)<br>2.78 (0.07-15.5)<br>4.43 (0.11-24.7)	Age, sex, year

CC: coloncancer, CRC: colorectal cancer, PSC: primary sclerosing cholangitis, RC: rectal cancer, UC: ulcerative colitis

Supplementary Table 8. Prospective studies of primary sclerosing cholangitis and total cancer

First author, publication year, country	Study name	Study period	Number of participants, number of cases	Exposure, subgroup	Comparison	Relative risk (95% confidence interval)	Adjustment for confounders
Bergquist A et al, 2002, Sweden	Swedish Cancer and Death registries	1970-1998 - 1998, 5.7 years follow-up	604 PSC patients, men and women, mean age 40.7 years: 87 cancer cases	PSC	Yes vs. no	6.1 (4.9-7.5)	Age, sex, calendar-time
Card TR et al, 2008, United Kingdom	UK General Practice Research Database	1991-2001 - 2001, ~3.1 years follow-up	223 PSC patients and 2217 controls, men and women, age 0-≥85 years: 128 cancer deaths	PSC	Yes vs. no	2.44 (1.53-3.88)	Age, sex, smoking status
de Valle MB et al, 2012, Sweden	Vastra Gotaland	1992-2005 - 2008, 6.5 years follow-up	199 PSC patients, men and women, age 18.2-76.8 years: 29 cancer cases	PSC	Yes vs. no	4.17 (2.79-5.99)	Age, sex
Ngu JH et al, 2012, New Zealand	Canterbury	1980-2008 - 2010, 7 years follow-up	81 PSC patients, men and women, age 17-82 years: NA cancer deaths<br>23 cancer cases	PSC, hepatobiliary cancer mortality<br>PSC, nonhepatobiliary cancer mortality<br>PSC, extrahepatic cancer mortality<br>PSC, total cancer incidence	Yes vs. no<br>Yes vs. no<br>Yes vs. no<br>Yes vs. no	116.9 (66.8-189.8)<br>1.5 (0.7-3.0)<br>1.5 (0.3-4.3)<br>5.2 (3.3-7.8)	Age, sex
Barner-Rasmussen N et al, 2020, Finland	Hospital District of Helsinki and Uusimaa (HUS)	1990-2015, ~25 years follow-up	632 PSC patients, mean age 40.6 years: 69 cancer cases<br>38 cancer deaths	PSC, incidence<br>PSC, mortality	Yes vs. no<br>Yes vs. no	3.39 (2.64-4.29)<br>5.92 (4.19-8.12)	Age, sex, year

PSC: primary sclerosing cholangitis

Supplementary Table 9. Prospective studies of primary sclerosing cholangitis and all-cause mortality

First author, publication year, country	Study name	Study period	Number of participants, number of cases	Exposure, subgroup	Comparison	Relative risk (95% confidence interval)	Adjustment for confounders
Card TR et al, 2008, United Kingdom	UK General Practice Research Database	1991-2001 - 2001, ~3.1 years follow-up	223 PSC patients and 2217 controls, men and women, age 0-≥85 years: 248 deaths	PSC	Yes vs. no	2.97 (2.19-4.02)	Age, sex, smoking status
de Valle MB et al, 2012, Sweden	Vastra Gotaland	1992-2005 - 2008, 6.5 years follow-up	199 PSC patients, men and women, age 18.2-76.8 years: 42 deaths	PSC	Yes vs. no	4.20 (3.01-5.69)	Age, sex
Ngu JH et al, 2012, New Zealand	Canterbury	1980-2008 - 2010, 7 years follow-up	81 PSC patients, men and women, age 17-82 years: 22 deaths	PSC	Yes vs. no	4.1 (2.6-6.3)	Age, sex
Boonstra K et al, 2013, Netherlands	Groningen, Rotterdam, Leiden	2000-2007 - 2012, 7.7 years follow-up	590 PSC patients, men and women, mean age 38.9 years: 97 deaths	PSC	Yes vs. no	4.2 (3.2-5.4)	Age, sex
Barner-Rasmussen N et al, 2020, Finland	Hospital District of Helsinki and Uusimaa (HUS)	1990-2015, ~25 years follow-up	632 PSC patients, mean age 40.6 years: 69 cancer cases<br>38 cancer deaths	PSC, incidence<br>PSC, mortality	Yes vs. no<br>Yes vs. no	3.39 (2.64-4.29)<br>5.92 (4.19-8.12)	Age, sex, year

PSC: primary sclerosing cholangitis

Supplementary Table 10. Prospective studies of primary sclerosing cholangitis and cardiovascular disease

First author, publication year, country	Study name	Study period	Number of participants, number of cases	Exposure, subgroup	Comparison	Relative risk (95% confidence interval)	Adjustment for confounders
Ngu JH et al, 2012, New Zealand	Canterbury	1980-2008 - 2010, 7 years follow-up	81 PSC patients, men and women, age 17-82 years: 22 deaths	PSC	Yes vs. no	0.5 (<0.1-2.9)	Age, sex
Ludvigsson JF et al, 2014, Sweden	Swedish Medical Registries	1970-2004, ~46.6 years follow-up	678 PSC patients and 6347 controls, men and women, age NA:<br>203/745 CVD cases<br>30/226 hypertensive disease cases<br>17/221 ischemic heart disease cases<br>13/31 pulmonary heart disease cases<br>20/134 cerebrovascular disease cases<br>17/34 cases of diseases of arteries, arterioles, capillaries<br>127/203 cases of diseases of veins, lymphatic vessels and lymph nodes	PSC, CVD<br>PSC, hypertensive disease<br>PSC, IHD<br>PSC, pulmonary heart disease<br>PSC, cerebrovascular disease<br>PSC, diseases of arteries, arterioles, capillaries<br>PSC, diseases of veins, lymphatic vessels, lymph nodes	Yes vs. no<br>Yes vs. no<br>Yes vs. no<br>Yes vs. no<br>Yes vs. no<br>Yes vs. no<br>Yes vs. no	3.34 (2.86-3.91)<br>1.56 (1.07-2.28)<br>0.90 (0.55-1.48)<br>5.03 (2.63-9.63)<br>1.74 (1.08-2.78)<br>5.61 (3.13-10.06)<br>6.95 (5.57-10.06)	Age, sex, follow-up duration, county

NA: not available, PSC: primary sclerosing cholangitis

Supplementary Table 11. Prospective studies of primary sclerosing cholangitis and other cancers

First author, publication year, country	Study name	Study period	Number of participants, number of cases	Exposure, subgroup, outcome	Comparison	Relative risk (95% confidence interval)	Adjustment for confounders
Bergquist A et al, 2002, Sweden	Swedish Cancer and Death registries	1970-1998 - 1998, 5.7 years follow-up	604 PSC patients, men and women, mean age 40.7 years: 1 stomach cancer case	PSC<br>PSC, excluding 1st year of follow-up	Yes vs. no<br>Yes vs. no	2.2 (0.1-12.5)<br>2.5 (0.1-14.1)	Age, sex, calendar-time
Ngu JH et al, 2012, New Zealand	Canterbury	1980-2008 - 2010, 7 years follow-up	81 PSC patients, men and women, age 17-82 years: 2 lung cancer cases<br>2 breast cancer cases<br>1 prostate cancer case<br>1 renal cancer case<br>3 nonmelanoma skin cancer cases	PSC, lung cancer<br>PSC, breast cancer<br>PSC, prostate cancer<br>PSC, renal cell cancer<br>PSC, nonmelanoma skin cancer	Yes vs. no<br>Yes vs. no<br>Yes vs. no<br>Yes vs. no	4.3 (0.5-15.4)<br>4.1 (0.5-14.8)<br>1.2 (<0.1-6.7)<br>0.8 (<0.1-4.6)<br>117.6 (24.2-343.6)	Age, sex
Barner-Rasmussen N et al, 2020, Finland	Hospital District of Helsinki and Uusimaa (HUS)	1990-2015, ~25 years follow-up	632 PSC patients, mean age 40.6 years: 4 gallbladder cancer cases<br>NA gallbladder cancer deaths	PSC, incidence<br>PSC, mortality	Yes vs. no<br>Yes vs. no	78.3 (21.3-200)<br>45.7 (5.53-165)	Age, sex, year

NA: not available, PSC: primary sclerosing cholangitis

Supplementary Table 12. Study quality of the included studies

Author, publication year, country	Representativeness	Selection of non-exposed cohort	Exposure assessment	Demonstration that the outcome was not present at the beginning of the study	Adjusted for one confounder	Adjusted for one additional confounder	Assessment of outcome	Long enough follow-up	Adequacy of follow-up	Total
Burak K et al, 2004, USA	1	0	1	0	1	1	1	1	0	6
de Valle MB et al, 2012, Sweden	1	0	1	0	1	1	1	1	0	6
Boonstra K et al, 2013, Netherlands	1	0	1	0	1	1	1	1	1	7
Barner-Rasmussen N et al, 2020, Finland	1	0	1	1	1	1	1	1	1	8
Bergquist A et al, 2002, Sweden	1	0	1	0	1	1	1	1	1	7
Ngu JH et al, 2012, New Zealand	1	0	1	0	1	1	0	1	1	6
Card TR et al, 2008, United Kingdom	1	1	1	0	1	1	1	0	0	6
Søgaard KK et al, 2014, Denmark	1	0	1	1	1	1	1	0	0	6
Liang H et al, 2017, United Kingdom	1	1	1	1	1	1	1	1	0	8
Loftus EV et al, 1996, USA	1	0	1	0	1	1	0	0	1	5
Ludvigsson JF et al, 2014, Sweden	1	0	1	0	1	1	1	1	0	6

Supplementary Figure 1. Influence analysis for primary sclerosing cholangitis and cholangiocarcinoma

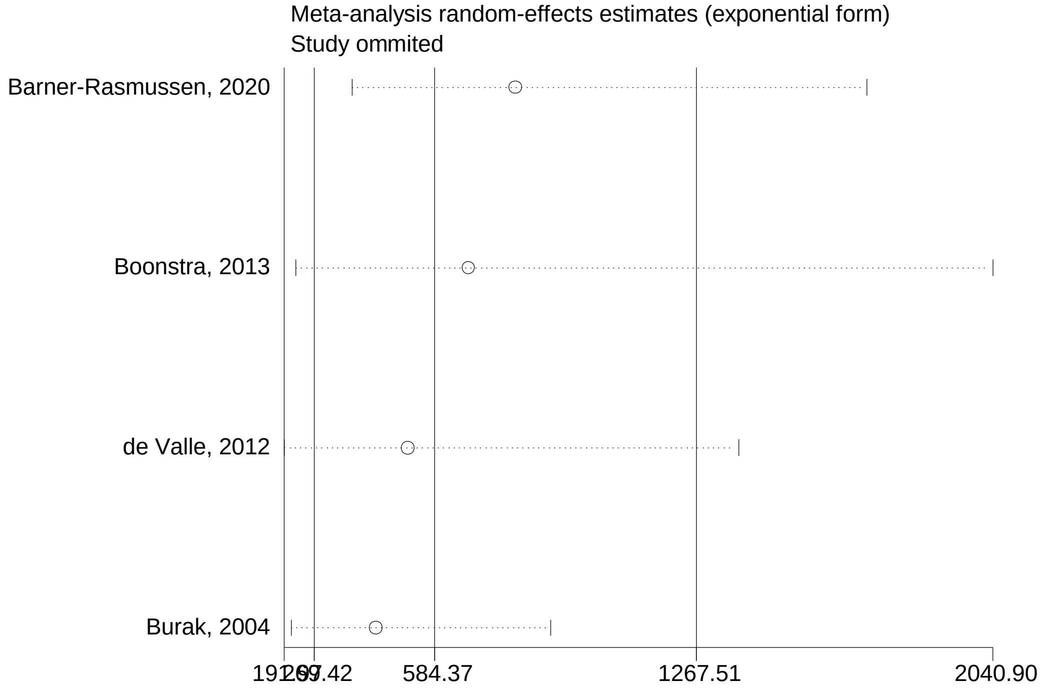

Study omitted	e^coef.	[95% Conf.	Interval]
Barner-Rasmussen, 2020	793.73743	368.17923	1711.1752
Boonstra, 2013	672.54633	221.62656	2040.9042
de Valle, 2012	514.20544	191.96768	1377.3528
Burak, 2004	430.35608	208.91249	886.526
Combined	584.37179	269.41827	1267.5101

Supplementary Figure 2. Influence analysis for primary sclerosing cholangitis and hepatobiliary cancer

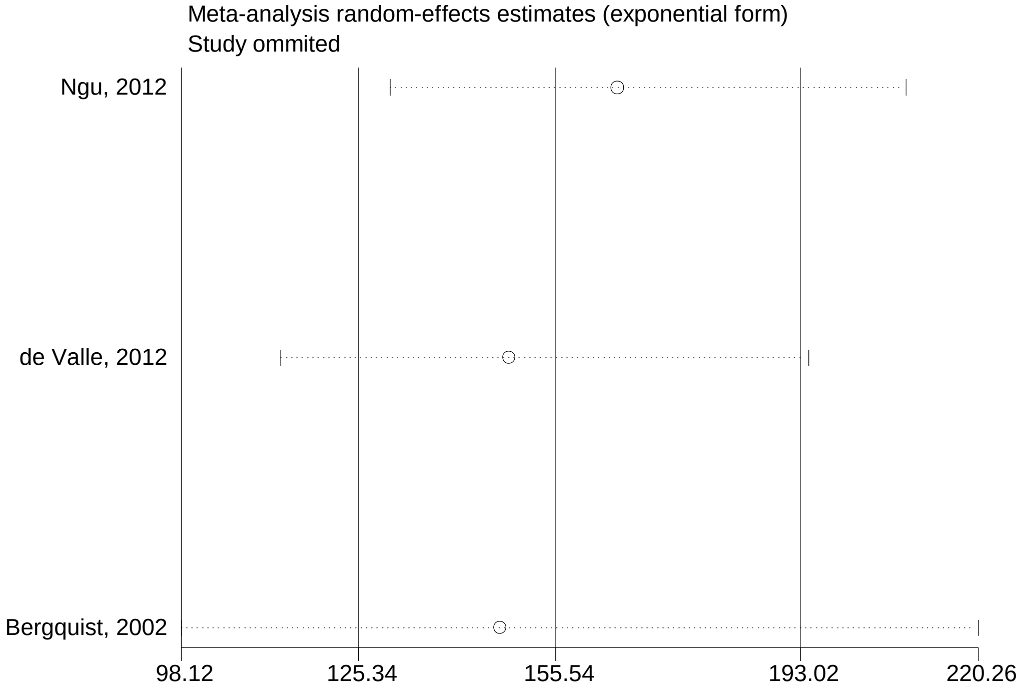

Study omitted	e^coef.	[95% Conf.	Interval]
Ngu, 2012	164.97865	130.15137	209.12538
de Valle, 2012	148.41177	113.4073	194.22078
Bergquist, 2002	147.01065	98.123169	220.25513
Combined	155.54197	125.33847	193.02378

Supplementary Figure 3. Influence analysis for primary sclerosing cholangitis and gastrointestinal cancer

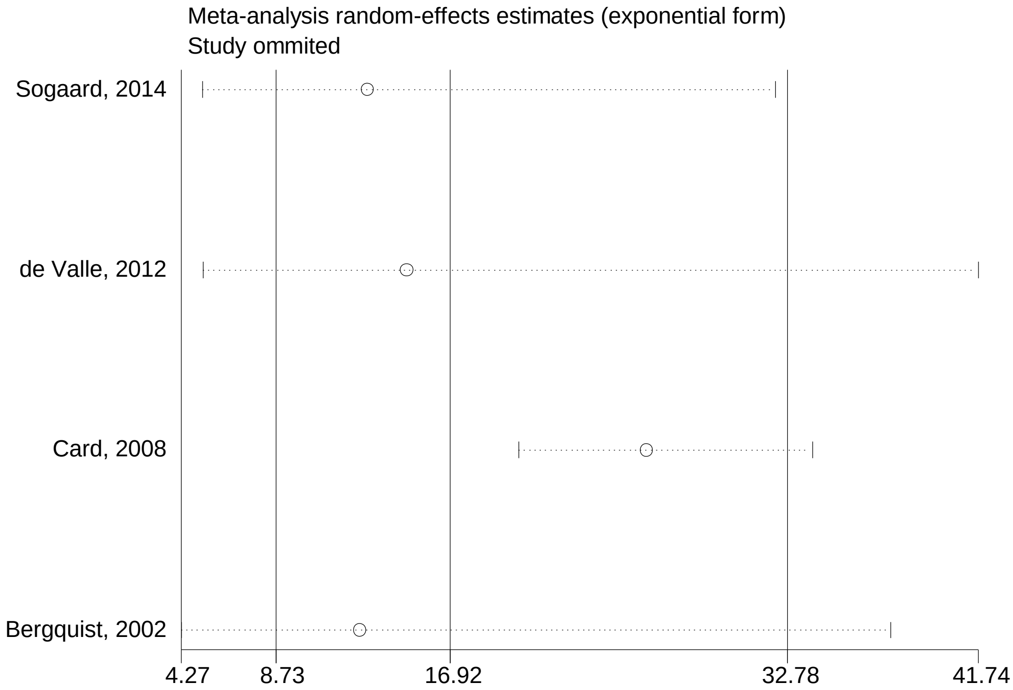

Study omitted	e^coef.	[95% Conf.	Interval]
Sogaard, 2014	13.032947	5.2757335	32.196033
de Valle, 2012	14.880298	5.3042088	41.744823
Card, 2008	26.153198	20.140051	33.961674
Bergquist, 2002	12.667599	4.2665243	37.610958
Combined	16.916358	8.7299996	32.779289

Supplementary Figure 4. Influence analysis for primary sclerosing cholangitis and pancreatic cancer

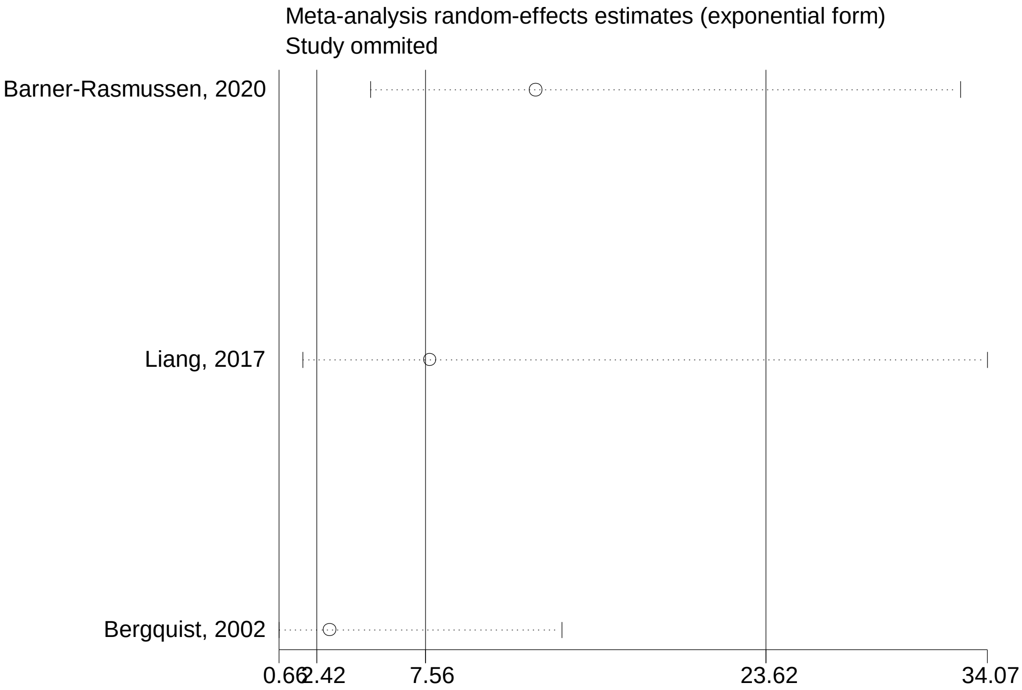

Study omitted	e^coef.	[95% Conf.	Interval]
Barner-Rasmussen, 2020	12.751542	4.9592934	32.7873
Liang, 2017	7.7758412	1.7747267	34.069302
Bergquist, 2002	3.0349834	0.65894431	13.978609
Combined	7.5580176	2.418183	23.622542

Supplementary Figure 5. Influence analysis for primary sclerosing cholangitis and colorectal cancer

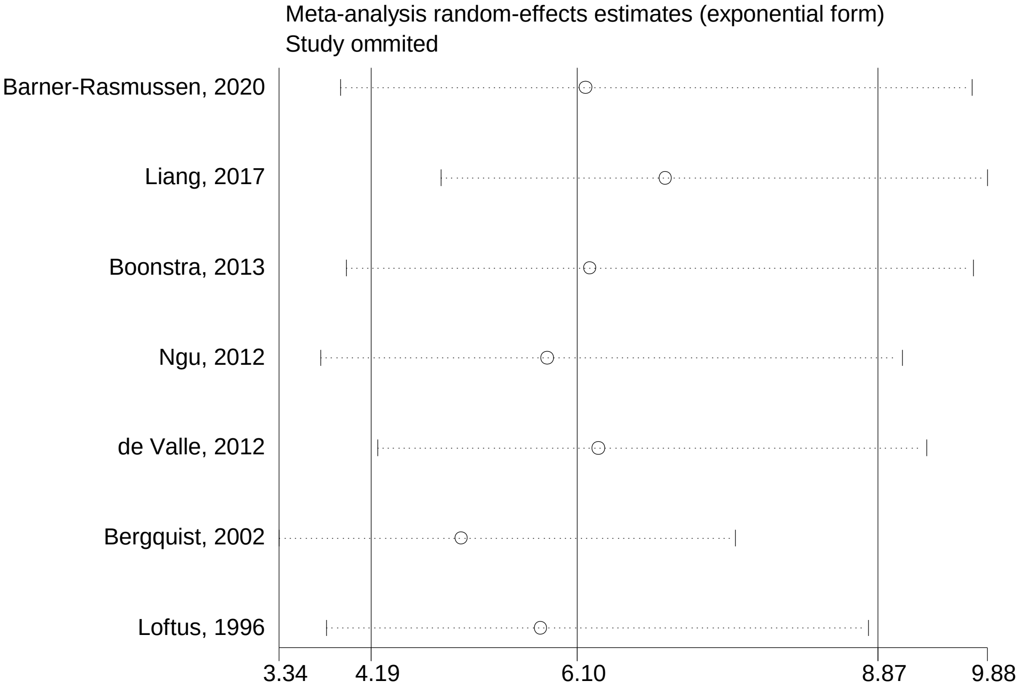

Study omitted	e^coef.	[95% Conf.	Interval]
Barner-Rasmussen, 2020	6.1707134	3.909867	9.7388735
Liang, 2017	6.91324	4.8358364	9.8830652
Boonstra, 2013	6.2153354	3.9611478	9.7523232
Ngu, 2012	5.8207803	3.7252512	9.0950861
de Valle, 2012	6.2932515	4.2502542	9.3182707
Bergquist, 2002	5.0258203	3.3444014	7.5525827
Loftus, 1996	5.7614946	3.7803471	8.7808924
Combined	6.0976913	4.1910404	8.8717445

Supplementary Figure 6. Influence analysis for primary sclerosing cholangitis and total cancer

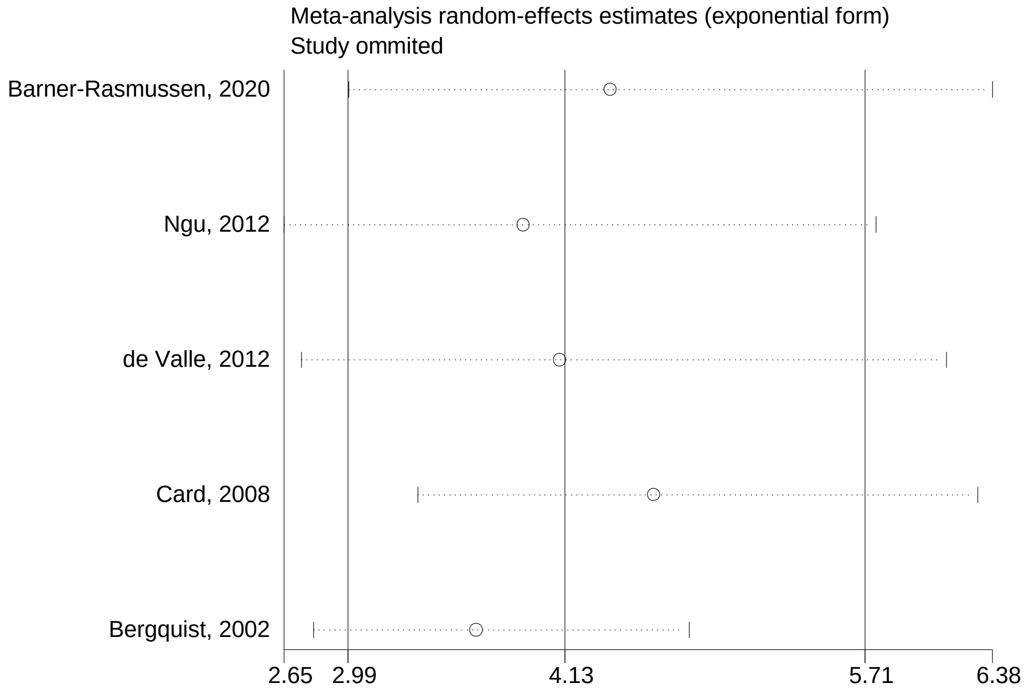

Study omitted	e^coef.	[95% Conf.	Interval]
Barner-Rasmussen, 2020	4.3701196	2.991837	6.3833504
Ngu, 2012	3.9119959	2.6528106	5.768867
de Valle, 2012	4.1029878	2.7417088	6.1401515
Card, 2008	4.5998974	3.3559885	6.3048658
Bergquist, 2002	3.6640389	2.8061283	4.7842364
Combined	4.1308989	2.987148	5.7125812

Supplementary Figure 7. Influence analysis for primary sclerosing cholangitis and all-cause mortality

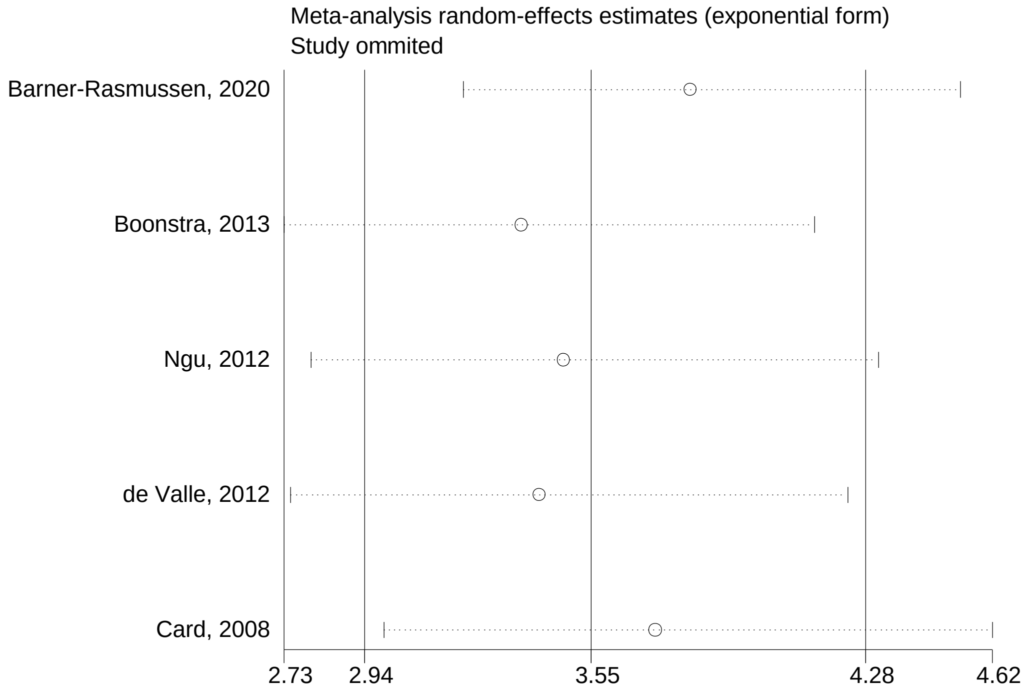

Study omitted	e^coef.	[95% Conf.	Interval]
Barner-Rasmussen, 2020	3.8129308	3.2046387	4.5366864
Boonstra, 2013	3.361927	2.7258489	4.1464348
Ngu, 2012	3.4747977	2.7963603	4.3178344
de Valle, 2012	3.4087121	2.7431645	4.2357354
Card, 2008	3.7189538	2.9919324	4.6226373
Combined	3.5487863	2.9409168	4.2822987
